# Microbial dynamics and metabolite changes in Chinese Rice Wine fermentation from sorghum with different tannin content

**DOI:** 10.1038/s41598-018-23013-1

**Published:** 2018-03-15

**Authors:** Jialiang Xu, Huijun Wu, Zhiwei Wang, Fuping Zheng, Xin Lu, Zhenpeng Li, Qing Ren

**Affiliations:** 1 Beijing Higher Institution Engineering Research Center of Food Additives and Ingredients, School of Food and Chemical Engeering, Beijing Technology and Business University, Beijing, 100048 China; 2 State Key Laboratory for Infectious Disease Prevention and Control, National Institute for Communicable Disease Control and Prevention, Chinese Center for Disease Control and Prevention, Beijing, 102206 China

## Abstract

Chinese rice wine (CRW) is the oldest kind of wine in China and is mainly fermented by wheat Qu and yeast with rice, millet, etc. This gives CRW a unique quality, but the flavor components are complex. Its formation is related to microorganisms, but the link between CRW and microorganisms is poorly understood. Here, we used two kinds of sorghum (JZ22 and JB3, of which JZ22 has a higher tannin content) as the raw materials to brew and determined the structural and functional dynamics of the microbiota by metagenomics and flavor analyses. We detected 106 (JZ22) and 109 (JB3) volatile flavor compounds and 8 organic acids. By correlation analysis, we established 687 (JZ22) and 496 (JB3) correlations between the major flavor compounds and microbes. In JZ22, *Blautia*, *Collinsella*, *Bifidobacterium*, *Faecalibacterium* and *Prevotella* had the most correlations with flavor production. In JB3, the top 5 genera were *Stenotrophomonas*, *Bdellovibrio*, *Solibacillus*, *Sulfuritalea* and *Achromobacter*. In addition, more esters were detected, and more microorganisms correlated with ester generation in JZ22. This study provides a new idea for the micro ecological diversity of CRW fermented with sorghum. This is of significance for improving the quality and broadening the CRW varieties.

## Introduction

Chinese rice wine (CRW), a traditional alcohol drink, is fermented from rice with wheat Qu and yeast. It is one of the oldest drinks in the world, along with beer and wine^1^. CRW is also called “liquid cake” because of its abundant nutrition and pleasant aroma^2^. Sorghum is a kind of natural and high-nutrition functional food that is rich in dietary fiber, protein, fat, folic acid, ferrum and other microelements. Sorghum is the main raw material for producing spirits. Chinese liquor brewed with sorghum, such as Maotai-flavored liquor, has long enjoyed a good reputation. However, with the changes in China’s policies on the liquor industry and the increase in people’s living standards, high-nutrition, low-alcohol CRWis becoming increasingly popular with consumers. Brewing CRW with sorghum not only increases the value of sorghum but also combines these two nutritious foods together.

Flavor differences can be caused by many factors, such as the raw ingredients used in the fermentation, fermentation conditions, distillation practices, and aging processes^3^. Xiong used 36 varieties of glutinous rice as brewing materials to study the influence of the nutrients in the raw materials on the wine yield of yellow rice wine^4^. Different raw materials also greatly affected the production of phenylethanol. Compared with rice as a raw material, more phenylethanol was produced when sorghum and maize were used. The L-phenylalanine content in the raw materials had a positive effect on B-phenylethanol yield^5^. The flavor compounds were mainly produced by biochemical reactions of microorganisms. Cui found through gene sequencing a kind of microorganism in liquor that produces pyrazine compounds^6^. Zhang identified the relationship between the flavor substances and aroma-producing microorganisms and performed fermentation by separating and screening Daqu microbes^7^. Fujii and others obtained mutant strains of yeast by molecular biological techniques and improved the isoamyl acetate content in sake^8^. Beaumont found that esterase is one of the most important enzymes in the synthesis and hydrolysis of volatile esters. This enzyme is produced by a series of microorganisms, such as *Aspergillus*, *Rhizopus*, *Bacillus*, *Lactobacillus*, *Wickerhamomyces* and *Saccharomycopsis*^9^. *Aspergillus* and *Bacillus* have been reported to be involved in amylase and acid protease production and can increase the amylase and acid protease activities in inoculated Daqu^10^. The research of De and others showed that some lactic acid bacteria (LAB) are considered to be main functional contributors in proteolysis, lipolysis, and amino acid/lipid catabolism^11^. In a Chinese rice wine research, a total of 64 volatile compounds were identified and quantified in Shaoxing rice wines^12^. The bacterial community structures and diversity of the Chinese rice wine varied significantly during different fermentation stages. *Lactobacillus* (the predominant genus) and *Bacillus* might take an active part in flavor development in CRW^13^. Lv and others evaluated the differences in the microbial communities between the “Qu” samples obtained from the two locations in China (Suzhou and Yichang). The microorganism community structures were significantly different^14^. That research was conducted solely on the analysis of the CRW’s flavor substances and microorganisms. However, the relationship between aroma compounds and bacterial community in CRW has not been fully reported. Therefore, we aim to study the effects of brewing raw materials and microorganisms on flavor compounds in Chinese rice wine.

To achieve this goal, this study monitored bacterial succession by high-throughput sequencing and the volatile compound dynamics by HS-SPME/GC–MS during the brewing process. The relationship between the bacterial community and the volatile compounds was analyzed. In this work, two kinds of sorghum were used. One of them is Jinza No. 22 (JZ22), whose tannin content is 1.57%, and the another is Jinbai No. 3 (JB3), with a tannin content of 0.02%. We would like to research the effect of tannin content on the microbial communities and flavor compounds in the brewing of yellow rice wine. The result will help in the development of CRW.

## Results

### Changes in acid, reducing sugar and alcohol

**Figure 1:**
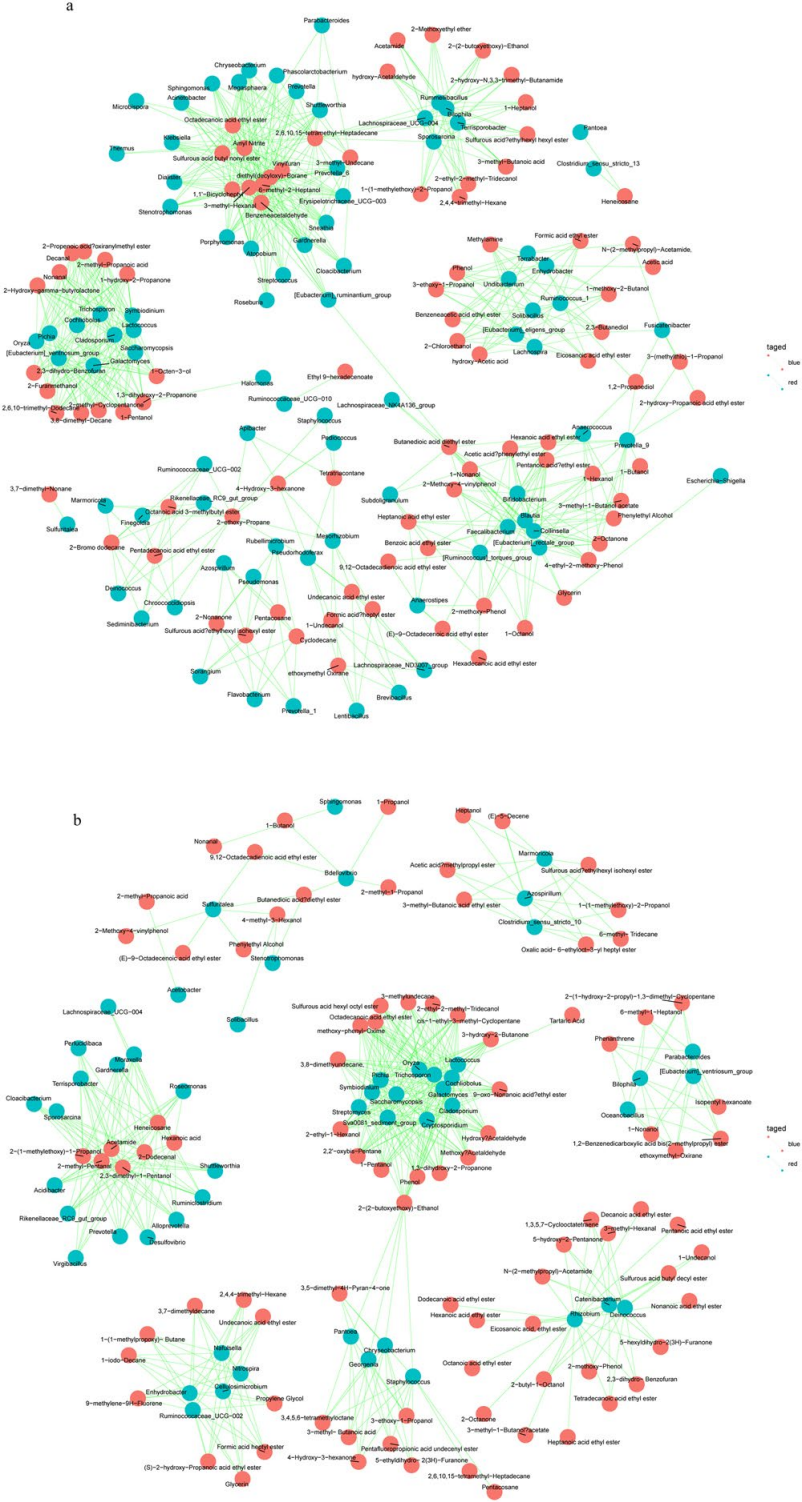
Changes in the acid, reducing sugar and alcohol found in JZ22 (**a**) and JB3 (**b**) Chinese rice wine brewed from sorghum during fermentation.

The data in Fig. 1 show that the concentration of acid in both JZ22 and JB3 continued to increase gradually, especially from the 4^th^ to 6^th^ days of fermentation, where there was a increase in JB3. It increased from the 6^th^ to 8^th^ days in JZ22. The alcohol continued to increase gradually and had rapid growth from the 2^nd^ to 4^th^ days in JZ22 and from the 4^th^ to 6^th^ days in JB3. From the 8^th^ to 12^th^ days, it increased gently. The concentration of the reducing sugar reached a peak value on the 2^nd^ day and decreased gradually in JZ22. However, it continued to fall in JB3. Detailed data are attached in Supplementary Table S1.

### Changes in organic acid during CRW fermentation

The organic acids identified in Chinese rice wine during fermentation were oxalic acid, tartaric acid, pyruvic acid, malic acid, α-ketoglutaric acid, lactic acid, citric acid and succinic acid (Table S2). In both JZ22 and JB3 samples, oxalic acid, tartaric acid, malic acid, lactic acid, citric acid and succinic acid have a continuing upward trend. Pyruvic acid reached the highest level at the 4^th^ day and then began to decline. However, the content of the pyruvic acid in JZ22 was much higher than JB3. α-Ketoglutaric acid fluctuated greatly, and the changes in the two samples were significantly different. It has been reported that tartaric acid, lactic acid, citric acid, and succinic acid were the primary acids, and the results of this experiment are in agreement with the previous report^15^.

### Analysis of volatile compounds in Chinese rice wine during

#### Fermentation

A total of 106 volatile compounds were detected in the 7 days’ samples of JZ22, including 16 alkanes, 24 alcohols, 32 esters, 12 ketones, 5 aldehydes, 5 acids, 4 phenols, and 8 other compounds (Table S3a). Among the compounds identified, heneicosane was the major alkane with a high concentration. Among the alcohols detected, 2-methyl-1-propanol, 3-methyl-1-butanol, glycerin, 2,3-butanediol and phenylethyl alcohol had the highest concentrations. Among the esters, hexanoic acid ethyl ester, octanoic acid ethyl ester, decanoic acid ethyl ester, dodecanoic acid ethyl ester, hexadecanoic acid ethyl ester, (E)-9-octadecenoic acid ethyl ester, 9,12-octadecadienoic acid ethyl ester, amyl nitrite and benzoic acid ethyl ester were the major esters with the highest concentrations. Among the ketones, 2-octanone and dihydro-5-propyl-2(3 H)-furanone were the predominant constituents. Among the phenols, 2-methoxy-4-vinylphenol and 2-methoxy-4-vinylphenol had a high relative concentration. On the last day, 47 compounds were detected, including 4 alkanes, 13 alcohols, 21 esters, 1 ketones, 2 aldehydes, 3 phenols, and 3 other compounds. In the 7 days’ samples of JB3, we identified 109 volatile compounds, including 16 alkanes, 26 alcohols, 34 esters, 9 ketones, 6 aldehydes, 4 acids, 4 phenols, and 10 other compounds (Table S3b). 2-Methyl-1-propanol, 3-methyl-1-butanol, glycerin, 2,3-butanediol, phenylethyl alcohol, hexadecanoic acid ethyl ester, (E)-9-octadecenoic acid ethyl ester, 9,12-octadecadienoic acid ethyl ester, amyl nitrite, 2-octanone, 4-ethyl-2-methoxy-phenol and 2-methoxy-4-vinylphenol were the major flavor compounds. On the last day, 47 compounds were detected, including 5 alkanes, 12 alcohols, 14 esters, 2 ketones, 1 aldehyde, 2 phenols, and 3 other compounds.

In comparing the volatile compounds of the two kinds of sorghum yellow rice wine, it can be found that there is not much difference in their quantity and species. However, in some flavor substances, JZ22 is much higher than JB3, such as 2-methyl-1-propanol, 3-methyl-1-butanol, glycerin, 2,3-butanediol, phenylethyl alcohol, octanoic acid ethyl ester, decanoic acid ethyl ester, 2-hydroxy-propanoic acid ethyl ester, tetradecanoic acid ethyl ester, hexadecanoic acid ethyl ester, 9,12-octadecadienoic acid ethyl ester and benzoic acid ethyl ester. They have a unique flavor and are often used in the preparation of flavors and fragrances. However, the 4-ethyl-2-methoxy-phenol and 2-methoxy-4-vinylphenol contents of JB3 were much higher. 4-Ethyl-2-methoxy-phenol has a pungent odor and is often used in perfumes, such as vanilla and whiskey^16^. Amyl nitrite was detected in both kinds of sorghum yellow wine and is not found in the CRW made from glutinous rice. Amyl nitrite lowers blood pressure and dilates blood vessels^17^.

#### Bacterial diversity during different fermentation stages

To explore the intra- and inter-variabilities in the bacterial community among the two kinds of fermentation materials, we estimated a series of alpha diversity indices using the t-test (Table S4). The same data shows that the difference between the early days and the last few days during the fermentation is significant. In comparing JZ22 with JB3, there was a remarkable difference from the 4^th^ to 8^th^ days, especially on the 8^th^ day.

The non-metric multidimensional scaling (NMDS) distribution based on the weighted UniFrac distance described the structures of the bacterial communities for all fermentation samples (Fig. S1). Although there was some overlap between JB3_D6 and JB3_D12, JZ22_D10 and JZ22_D0, there was a distinct separation between the samples of different time points.

**Figure 2:**
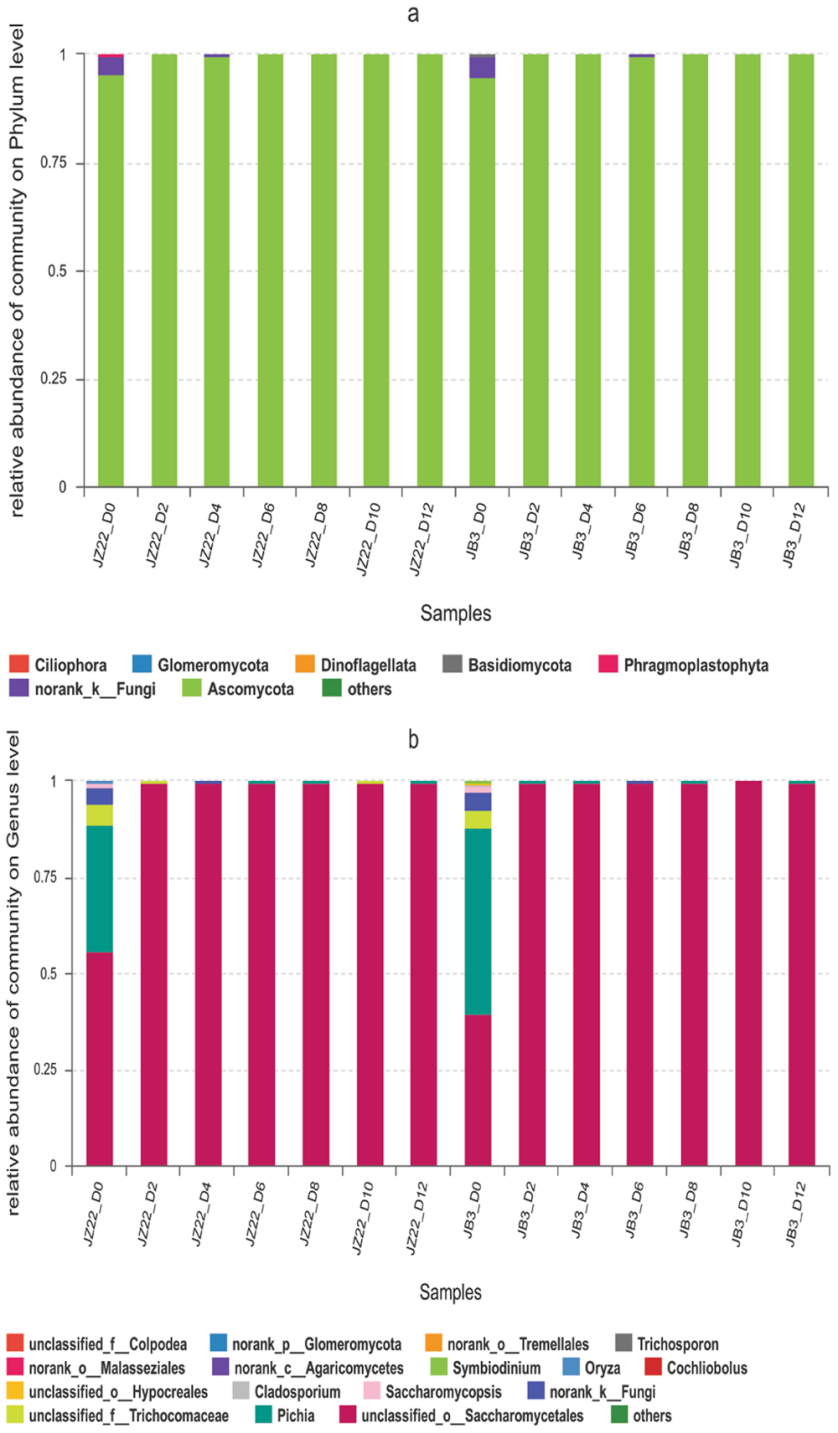
Relative abundance levels of bacterial phyla (**a**) and genera (**b**) during different fermentation stages of JZ22 and JB3.

Of all the total reads obtained from the 21 samples of JZ22, 1 059 218 reads passed the quality control filters. A total of 13 bacterial phyla, 23 classes, 50 orders, 74 families, 115 genera and 155 species were identified by matching the sequences of known bacteria. The results showed that, at the phylum level (Fig. 2a), *Firmicutes* was predominant in all samples, followed by *Cyanobacteria* and *Actinobacteria*. At the genus level (Fig. 2b), *Bacillus* was the most dominant genera, ranging from 83.17% to 94.86% of the total 16 S rRNA gene sequences. *Weissella* was second only to *Bacillus*, peaked on the 4^th^ day, and then began to decrease. In the early days of fermentation, *Paenibacillus* decreased up until the 10^th^ day.

In the JB3 samples, 1 050 616 high-quality sequences were obtained with Illumina MiSeq analysis. The 16S rRNA gene sequences indicated that 153 species of bacteria participated in the fermentation process and were distributed among the 13 phyla, 24 classes, 47 orders, 73 families and 119 genera. At the phylum level (Fig. 2a), same as JZ22, Firmicutes, Proteobacteria and Actinobacteria were the top three most abundant. *Bacillus* occupied the main position at the genus level (Fig. 2b). *Brevibacillus* was the second most dominant genus, and it increased and appeared to peak on the 8^th^ day before slightly decreasing at the end of fermentation. *Weissella* followed *Brevibacillus* and peaked on the 4^th^ day.

The bacterial community composition of JZ22 and JB3 was not distinguished at the phylum and family levels, but their changing trends were completely different. At the genus level, *Bacillus* in JZ22 showed a lower abundance on the 4^th^ day. However, in JB3, *Bacillus* fell to the lowest level on the 8^th^ day. Genus *Brevibacillus* played an important role in JB3; however, the proportion of *Brevibacillus* is very small in JZ22. Ethyl alcohol was the precursor of ethyl ester, and the content of ethyl ester in JZ22 was obviously higher than that in JB3, which may be related to the amount of *Weissella*^13^. Detailed information on the composition of bacteria is shown in the attached Table S5.

#### KEGG annotation exposes metabolic differences between JZ22 and JB3 fermentation

**Figure 3:**
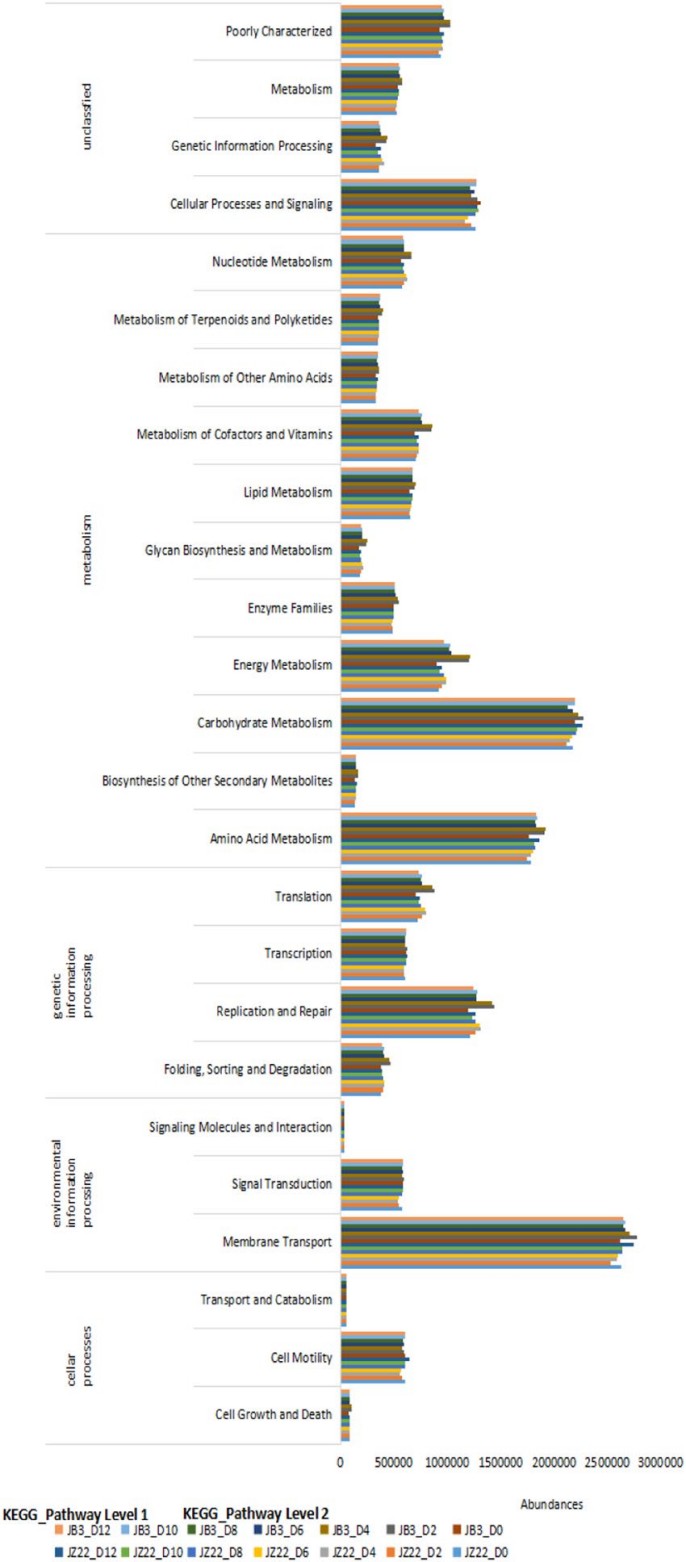
Relative abundance levels of predicted gene functions related to KEGG pathways at levels 1 and 2.

Based on the 16 S rRNA gene sequencing data, PICRUSt was used to predict the function of the microorganisms in the fermented mash, which showed similar gene functions among all 42 samples that were categorized into the 39 level-2 KEGG pathways (Fig. 3). Membrane Transport, Carbohydrate Metabolism,Amino Acid Metabolism, Replication and Repair, Cellular Processes and Signaling, Energy Metabolism, Poorly Characterized, Translation, Metabolism of Cofactors and Vitamins were the dominant KEGG pathways. We found that on day 0, JZ22 was similar to JB3, and there were differences in the metabolism during fermentation, especially in the later stage of fermentation. During fermentation, the ABC transporters, glycolysis/gluconeogenesis and pyruvate metabolism pathways had the highest abundance. Detailed information of the predicted gene functions related to KEGG pathways at levels 3 is shown in the attached Table S6.

#### Fungal diversity during different fermentation stages

**Figure 4:**
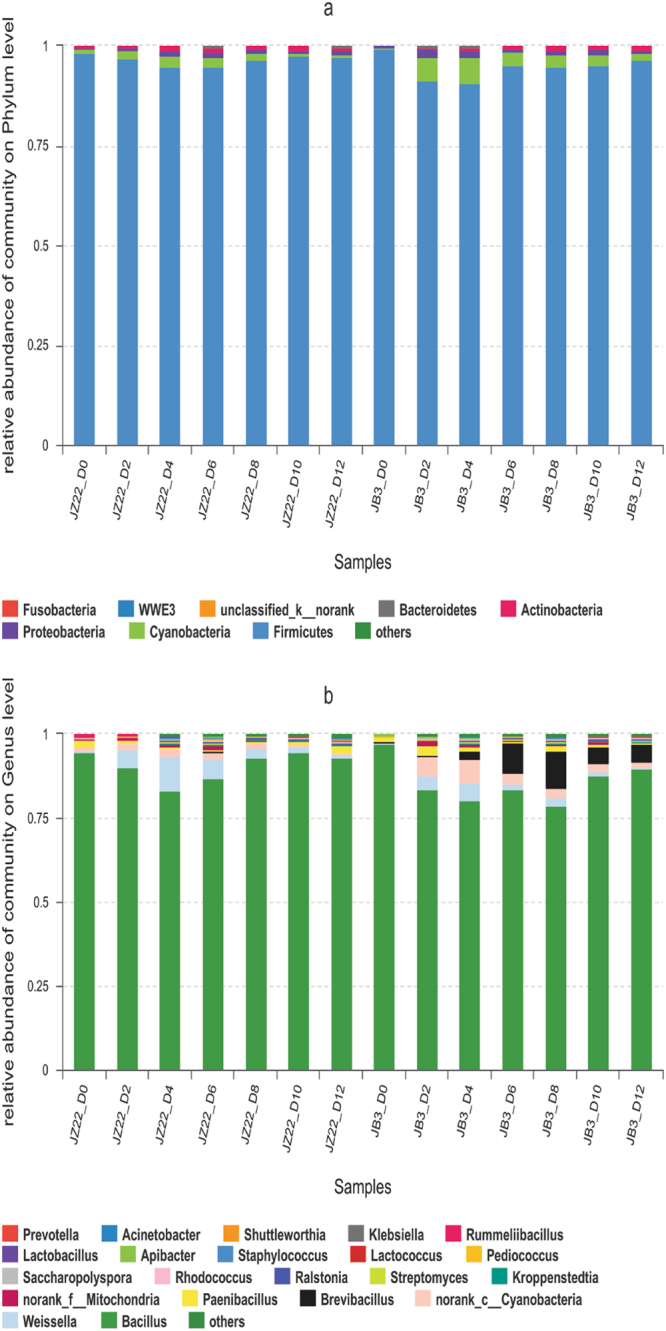
Relative abundance levels of fungi phyla (**a**) and genera (**b**) during different fermentation stages of JZ22 and JB3.

Regarding the fungal communities’ sequences in JZ22, 1 073 598 reads passed the quality control filters. Ten phyla, 17 classes, 18 orders, 21 families, 21 genera and 23 species were identified. At the phylum level (Fig. 4a), approximately 99% of the ITS region sequences could be assigned to the phylum Ascomycota. At the genus level (Fig. 4b), Unclassified Saccharomycetales was the most abundant through the whole fermentation process. *Pichia* decreased until 8th day and then increased in JZ22. The deep sequencing of the fungal communities obtained a total of 1 085 155 sequences in JB3, including 6 phyla, 12 classes, 13 orders, 17 families, 17 genera and 17 species. At the phylum level (Fig. 4a), Ascomycota was the most dominant phylum, accounting for more than 97.44% (94.94–99.93%). At the genus level (Fig. 4b), Unclassified Saccharomycetales was the most abundant, and *Pichia* had a sharp drop on the second day and then remained stable. The main fungi in the two CRW were almost the same, but at the genus level, *Cladosporium* and *Trichosporon* were only detected in JB3. Detailed information on the composition of fungal is shown in the attached Table S7.

#### Correlation between microorganisms and flavor compounds

The 28 main flavor substances in the fermentation process were correlated with the change in the microorganisms. In JB3, 44 microorganisms were involved in the production of the major flavor compounds (P < 0.05). Among them, *Stenotrophomonas*, *Bdellovibrio* and *Solibacillus* were related to more than 10 flavor compounds. In JZ22, there were 68 kinds of microorganisms associated with the formation of the major flavor substances (P < 0.05). *Blautia*, *Collinsella*, *Bifidobacterium*, *Faecalibacterium*, *Eubacterium rectale* group, *Ruminococcus torques* group, *Anaerococcus*, *Prevotella_9*, *Anaerostipes*, and *Escherichia-Shigella* were related to more than 15 flavor compounds. *Achromobacter*, *Eubacterium_ruminantium_group*, *Lachnospiraceae_NK4A136_group*, *Subdoligranulum*, *Bacteroides*, *Chryseobacterium*, *Roseburia* and *Sphingomonas* were involved in the formation of 10 to 15 flavor substances. Through association analysis, we can see that there were obviously more main flavor substances formed in the JZ22 brewing process than JB3.

**Figure 5:**
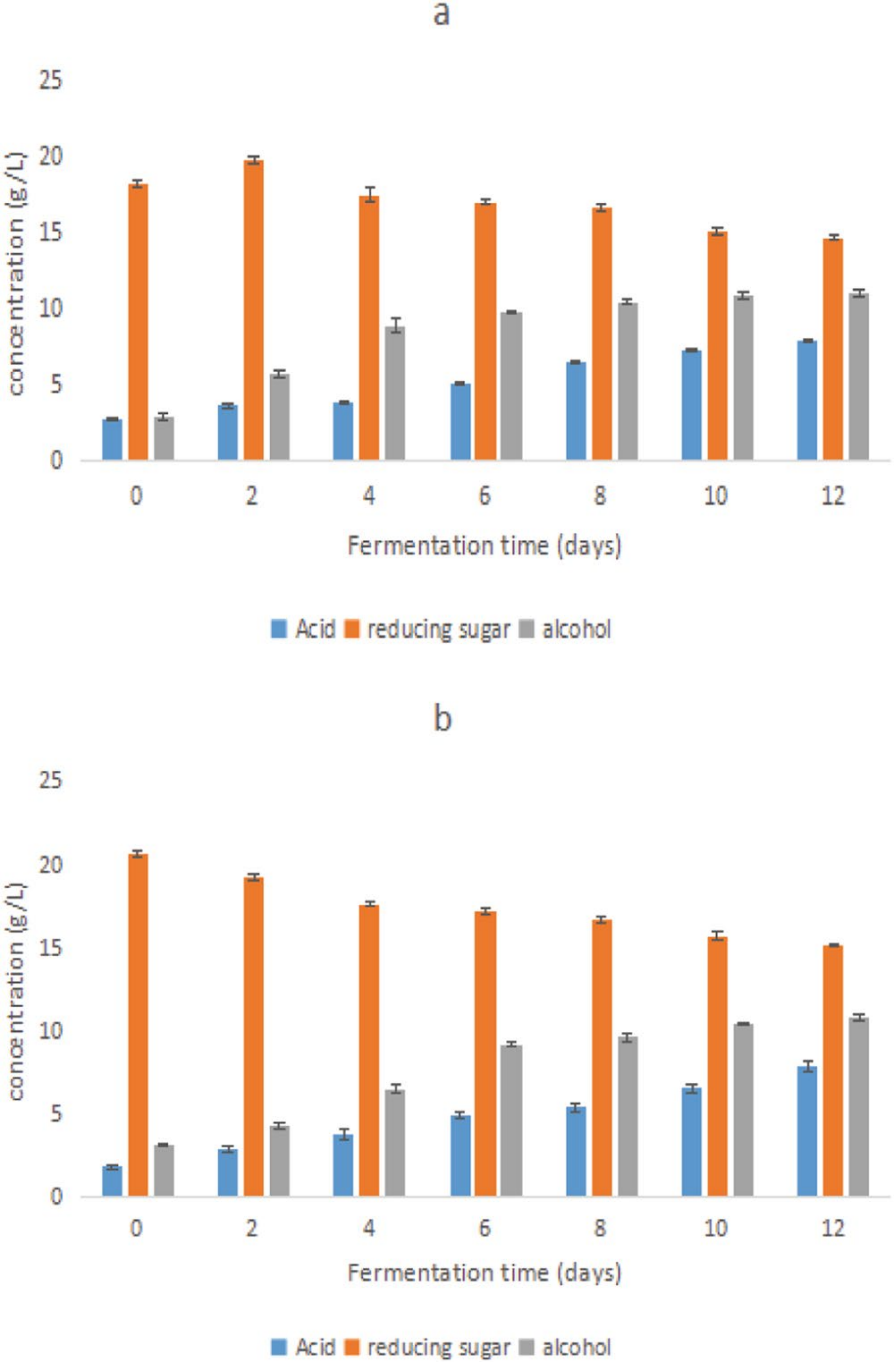
Correlation between microorganisms and flavor compounds in JZ22 (**a**) and JB3 (**b**).

The P value of the correlation analysis of all the flavor compounds and microorganisms was adjusted. We selected a Padjust <0.05 for the data (see Fig. 5). There were 687 correlations in JZ22. Among them, there were 20 alcohols, 24 esters and 3 phenols with a Padjust <0.05, including glycerin with *Bifidobacterium*; octadecanoic acid ethyl ester with *Lactococcus*, *Saccharomycopsis* and *Pichia*; acetic acid phenylethyl ester with *Blautia*; amyl nitrite with a complex series of microorganisms; and 2-methoxy-4-vinylphenol and 4-ethyl-2-methoxy-phenol with *Bifidobacterium*. In JB3, we established 496 correlations. These included 19 alcohols, 24 esters and 3 phenols. For example, octadecanoic acid ethyl ester had a closer correlation with *Cryptosporidium*, *Trichosporon*, *Galactomyces*, *Symbiodinium*, *Cladosporium*, *Pichia*, *Cochliobolus*, *Saccharomycopsis*, *Lactococcus* and *Rhizobium*. Tetradecanoic acid ethyl ester’s correlation with *Catenibacterium* and *Deinococcus* met the conditions. The correlation between *Lactococcus* and 3-hydroxy-2-butanone satisfied a Padjust <0.05. These correlations have not been reported in other reports and require further experimental verification.

## Discussion

In the fermentation process of Chinese rice wine, microorganisms play an important role in the formation of endogenous factors. Comparing the changes in the reducing sugar, total acid and alcohol content with the microbial flora, we found that the variation trends of total acid were similar to *Bacillus*, which has strong protease, amylase and lipase activity and can produce rich metabolic products and synthesize many organic acids.

Flavor differences may be caused by a number of factors, such as fermentation materials, fermentation conditions and aging processes. Southern rice wine is mostly fermented with glutinous rice, but this experiment used sorghum, which is usually used as raw material in the production of liquor. There are some reports about volatile compounds of Chinese rice wine, and most of them have used headspace solid-phase microextraction followed by gas chromatography–mass spectrometry^18^. A total of 64 volatile compounds were identified and quantified in Shaoxing rice wines. Xu and others determined the formation of volatiles in the enzymatic extrusion process of rice wine and confirmed its characteristic flavor compounds^1^. In Shaoxing rice wine, 10 bacterial genera, including *Bacillus* and *Lactobacillus*, were found to be predominant^12^. Similarly, five *Bacillus* species and three lactic acid bacteria were found to be the dominant bacteria in Hong Qu rice wine, a popular Chinese rice wine^19^. These microorganisms play important roles in fermentation and the production of special flavors^20^. Liu’s investigation showed that *Bacillus*, *Leuconostoc*, *Lactococcus*, *Weissella*, *Thermoactinomyces*, *Pseudomonas*, *Saccharopolyspora*, *Staphylococcus*, *Enterobacter* and *Lactobacillus* were the dominate genera during the Shaoxing rice wine fermentation process^12^. Although the main flora in this experiment are similar to those in Shaoxing rice wine, the flavor compounds are different. One hundred and six kinds of flavor compounds were detected in JZ22, and 109 were detected in JB3, which are more than in Shaoxing rice wine. This indicated that sorghum could produce more kinds of flavor compounds as raw materials. The two kinds of sorghum used in this experiment were fermented using the same conditions (high tannin content in the JZ22), and the Wheat Qu used the same conditions as well, but the content of the flavor compounds was different. This shows that the difference in the nutrients of the raw materials will affect the flavor of the final wine, which is largely caused by micro ecological changes and microbial metabolism during the fermentation process. In this experiment, JZ22 and JB3 had little difference in the main kinds of esters, but the content of the esters in JZ22 was higher than that in JB3. Through association analysis, we can see that the number of microbial genera involved in the formation of various esters in JZ22 was 2–3 times that of JB3, and the tannin content in JZ22 was also higher than JB3. We can estimate that tannins have important effects on the brewing process, microbial growth and the production of incense. According to previous reports, tannase (tannin acyl hydrolase, EC 3.1.1.20) catalyzes the hydrolysis of ester linkages in hydrolysable tannins and produces glucose and gallic acid^21^. Although gallic acid is widely distributed in nature, it is difficult for bacteria to use it as a carbon source for growth at neutral or alkaline pH. Only bacteria of the genera *Pseudomonas*^21^, *Lactobacillus*^22^ and *Sphingomonas*^23^ have been reported to be able to utilize free gallic acid as the sole carbon and energy source under aerobic conditions. Through correlation analysis, we found that *Sphingomonas* was related to the formation of 11 flavor substances in the JZ22 sample and was related to 6 flavor substances in JB3. These results indicate that *Sphingomonas* may play an important role in the degradation of tannins during the sorghum fermentation process, and the degradation products can produce a variety of aromatic compounds. The aerobic metabolism of gallic acid usually starts with a direct ring-cleavage reaction and the formation of the central intermediate, 4-oxalomesaconic acid, which then undergoes hydration to 4-carboxy-4-hydroxy-2-oxoadipic acid and aldol cleavage to oxaloacetic and pyruvic acids^24^. As determined by HPLC, the final pyruvate content in JZ22 was 14.09 mg/l, which was much higher than the 1.17 mg/l in JB3. Oxaloacetic and pyruvic acids are an important part of the TCA cycle and microbial carbon metabolism and may stimulate more microorganism involvement in the formation of the aroma. This also explains why the association analysis showed that JZ22 had more microorganisms involved in the formation of the flavor prediction. As seen in Fig. 4, in addition to *Bacillus*, *Weissella* occupied the most dominant position at the genus level in JZ22, while *Brevibacillus* occupied the dominate position in JB3. This may reveal that lactic acid bacteria (LAB) play a role in the metabolism of tannins. Therefore, in the process of brewing, tannins can be added for fermentation in order to increase the flavor of rice wine.

In both JZ22 and JB3, the characteristic flavor substances were 2-methyl-1-propanol, 3-methyl-1-butanol, phenethyl alcohol, decanoic acid ethyl ester, octanoic acid ethyl ester, 9,12-octadecadienoic acid ethyl ester and hexadecanoic acid ethyl ester, which is similar to the conclusion of the research on the volatile flavor substances of yellow rice wine^25^.

2-Methyl-1-propanol can be used in the preparation of flavors and fragrances and can also be used as a food flavoring agent. In the association of 2-methyl-1-propanol with microorganisms, it was found that the production of 2-methyl-1-propanol in JZ22 was related to *Ralstonia*, but no association was found in JB3. Studies have shown that *Ralstonia* can utilize H_2_O, CO_2_ and formic acid to generate 2-methyl-1-propanol^26^. At the end of fermentation, the content of 2-methyl-1-propanol in JZ22 was about two times that of JB3, which may be related to *Ralstonia* in the JZ22 fermentation system. The content of isobutyl alcohol may be controlled by controlling *Ralstonia*. Organic acids and other substances constitute the unique aroma of CRW; A proper amount of organic acids can harmonize the taste of yellow rice wine and stabilize the aroma, which will help to enrich and improve the aroma and quality of Chinese rice wine^27^. In addition, organic acids play an important role in improving intestinal function, preventing fatigue and other health functions. For example, malic acid functions in preventing fatigue, protecting the liver and strengthening heart function^28^, while citric acid functions in delaying senility, eliminating fatigue and lowering blood pressure^29^. Through association analysis, we found that *Lactococcus*, *Saccharomycopsis*, *Cladosporium*, *Fusicatenibacter*, *Cochliobolus*, and *Pichia* were related to the production of a variety of organic acids. Among them, *Lactococcus* mainly is involved in citric acid and lactic acid formation. *Saccharomycopsis* can ferment galactose, glucose, maltose and other sugars to produce acid. *Cladosporium* can lead to the decay of plants, such as wheat and grapes, and affects the flavor of wine^30^. *Fusicatenibacter* ferments glucose to produce lactic acid, formic acid, acetic acid, and succinic acid^31^. By controlling the related microorganisms, the organic acid content in the fermented mash can be adjusted properly, and the final aim of improving the quality of rice wine can be achieved.

The volatile flavor compounds in CRW are mainly derived from esters, and the production of esters comes from the following aspects: raw materials, esters and the combination of the fatty acids and alcohols in wheat Qu; the organic acids and amino acids combined with alcohols; and the decomposition, binding and oxidation of rice wine during aging. The aging process of yellow rice wine under natural conditions is quite slow, so the flavor compounds are mainly produced by biochemical reactions during fermentation. In the process of fermentation, the main esters are ethyl esters. The whole aroma of Chinese rice wine mainly depends on the aroma of esters. Ester material mainly comes from the esterification of acids and alcohols, as well as the activities of yeasts and other microorganisms in the brewing process. In addition to ethyl ester, this experiment also detected the amyl nitrite, which was not detected in southern rice wine. Amyl nitrite is a nitrous acid that can relax the vascular smooth muscle, especially the coronary vessels and cerebrovascular vessels, which make the blood vessels dilate and the blood pressure drop^32^. In JZ22, many more microorganisms were associated with amyl nitrite than in JB3, but both *Stenotrophomonas* and *Roseburia* were related to the formation of this compound in both CRWs. *Stenotrophomonas* can use succinate, malate, citrate, acetate, lactate and propionate as a growth factor^33^. *Roseburia* ferments carbohydrates such as glucose, maltose, sucrose, and starch and mainly produces butyric acid. Organic acids can be combined with alcohols to produce esters, and the relationship between these two microorganisms and amyl nitrite requires further study. Correlation is not always causation. As such, the relationship between microorganisms and the flavor substances in Chinese rice wine fermentation is very complex. The generation of a flavor substance may be the result of the synergetic effects of microorganisms.

The OTU abundance table was standardized by PICRUSt according to the KEGG database. In addition, for the pathway, 3 levels of information on metabolic pathways were obtained using PICRUSt, and the abundance tables at each level were obtained. In the high-expression metabolic pathways, ABC transporters are members of a transport system superfamily that is one of the largest and possibly one of the oldest families, with representatives in all extant phyla from prokaryotes to humans^34^. ABC transporters take up a large variety of nutrients, biosynthetic precursors, trace metals and vitamins, while exporters transport lipids, sterols, drugs, and a large variety of primary and secondary metabolites^35^. Glycolysis/gluconeogenesis is a series of reactions that degrade glucose and glycogen to pyruvate and ATP. It is a ubiquitous pathway of glucose degradation in all living organisms. Most monosaccharides, such as sugar and galactose, can be converted into one of these intermediates. Intermediates can then be used directly. Metatranscriptomic analysis revealed pyruvate metabolism in yeasts (genera *Pichia*, *Schizosaccharomyces*, *Saccharomyces* and *Zygosaccharomyces*) and lactic acid bacteria (genus *Lactobacillus*), which represented two stages in the production of flavor components, with the genera *Schizosaccharomyces* and *Lactobacillus* serving as the core functional microorganisms^36^. Specific expression of genes in metabolism also requires subsequent omics analysis.

In summary, this study is the first to reveal the differences between different sorghum-fermented CRWs and the microorganisms that are related to the production of the main flavor compounds. In addition, we can also deduce that tannins can affect the microbial structure, degrading themselves to produce more ester. Through the association of flavor substances and microbial fermentation process analysis, we can deduce the fermentation of the core microbiota. On the other hand, we target to produce important aromas and health ingredient strains. This provides a theoretical basis for the brewing of CRW with sorghum.

## Materials and Methods

### Sample collection

Samples were collected in Zhangjiakou city in the Hebei Province in April 2017. The fermentation was performed at a constant temperate of 28 °C and over a period of 12 days. Forty-two samples from different fermentation days were collected. Each sample was divided into two parts: one part was kept at 4 °C immediately after collection for flavor testing, and the other was transferred to sterile bottles and stored at −80 °C for DNA extraction.

### Determination of acid, reducing sugar, alcohol and flavor compounds

The DNS method was adopted to determine the reducing sugar content using glucose as reference standard. A 1.0 mL liquid sample was ten times diluted. Then, 1.0 mL of the dilution was shaken well with 1.0 mL of DNS solution and put in a boiling water bath for 5 min. When it cooled to room temperature, the mixture was diluted by water to a final volume of 10.0 mL, and its absorbance at 540 nm was read^37^. The alcohol content and acidity were measured according to the standard of GB/T13662-2008 and used to evaluate wine quality.

The flavor compounds and organic acids in the samples were analyzed by HS-SPME/GC-MS and HPLC, respectively. For HS-SPME-GC-MS analysis, each wine sample (8 mL) was placed in a 15 mL SPME glass vial together with 2.5 g of sodium chloride. The vial was tightly capped, and the extraction head (50/30 μm DVB/CAR/PDMS) was inserted. The vial was then placed in a water bath with ultrasonic waves for 45 min at 50 °C. After extraction, the fiber was introduced into the injection port of the GC-MS system (at 230 °C for 5 min), and the analytes from the fiber were thermally desorbed. Identification was carried out using a Shimadzu-QP2010 Plus-GCMS. Each concentrated fraction was analyzed on a DB-wax column (30 m × 0.25 mm i.d., 0.25 µm film thickness). The carrier gas, helium, was circulated at 1 mL/min in split-flow mode, and the split ratio was 50/1. The oven temperature program was as follows: 35 °C for 4 min; 5 °C/min ramp to 150 °C and holding for 2 min; and 3 °C/min ramp to 210 °C. The injector and detector temperature was 230 °C, and the ion source temperature was set 200 °C. The ion energy for electron impact (EI) was always 70 eV. The chromatograms were recorded by monitoring the total ion currents in the 30–350 mass range. Semi-quantification of the volatile compounds was performed using 2-octanol as the internal standard^16^. For HPLC analysis, each wine sample (5 mL) was placed in a tube, centrifuged at 10,000 r/min for 15 min, and then filtered through a 0.45 mm microporous membrane. Chromatographic conditions were based on the method described by Ye *et al*.^38^. The determination of organic acids was carried out by HPLC. The separations were carried out on an Agilent 1260 Infinity II equipped with a 250 mm × 4.6 mm and a 5 μm welch ultimate XB-C18 column. The column temperature was set at 30 °C. A mixture of phosphate buffer (0.01 mol/L (NH_4_)_2_HPO_4_), adjusted with phosphoric acid solution to pH 3.0, was used as the mobile phase with a flow rate of 0.8 mL/min. The detection wavelength was 215 nm.

### DNA extraction, PCR amplification and Illumina MiSeq sequencing

Microbial DNA was extracted from the samples using the E.Z.N.A.® soil DNA kit (Omega Bio-Tek, Norcross, GA, U.S.A.) according to the manufacturer’s protocols. The final DNA concentration and purification were determined by NanoDrop 2000 UV-vis spectrophotometer (Thermo Scientific, Wilmington, USA), and DNA quality was checked by 1% agarose gel electrophoresis. The V3-V4 hypervariable regions of the bacterial 16S rRNA gene were amplified by polymerase chain reactions (95 °C for 5 min; 25 cycles of 95 °C for 30 s, 55 °C for 30 s and 72 °C for 40 s; and a final extension at 72 °C for 10 min) with primers 338 F (5′-ACTCCTACGGGAGGCAGCAG-3′) and 806 R (5′-GGACTACHVGGGTWTCTAAT-3′)^39^ by a thermocycler PCR system (GeneAmp 9700, ABI, USA). For fungi ITS1 region was amplified by PCR (95 °C for 2 min; 30 cycles at 95 °C for 30 s, 61 °C for 30 s, and 72 °C for 45 s; and a final extension at 72 °C for 10 min) with the ITS1F (5′-AxxxCTTGGTCATTTAGAGGAAGTAA-3′) and ITS2 (5′-BGCTGCGTTCTTCATCGATGC-3′)^40^ primers. PCR products were purified according to a previous study^41^. Purified amplicons were pooled in equimolar ratios and paired-end sequenced (2 × 300) on an Illumina MiSeq platform (Illumina, San Diego, USA) according to standard protocols by Majorbio Bio-Pharm Technology Co. Ltd. (Shanghai, China).

### Processing of sequencing data

Raw fastq files were demultiplexed, quality filtered by Trimmomatic and merged by FLASH with the following criteria: (i) The reads were truncated at any site receiving an average quality score <20 over a 50 bp sliding window. (ii) Primers were matched exactly, allowing 2 nucleotide mismatches, and the reads containing ambiguous bases were removed. (iii) Sequences whose overlap was longer than 10 bp were merged according to their overlapping sequence^42^.

Operational taxonomic units (OTUs) were clustered with a 97% similarity cutoff using UPARSE (version 7.1 http://drive5.com/uparse/), and chimeric sequences were identified and removed using UCHIME. The taxonomy of each 16 S rRNA gene sequence was analyzed by an RDP Classifier algorithm (http://rdp.cme.msu.edu/) against the Silva (SSU123) 16 S rRNA database using a confidence threshold of 70%. The taxonomy of each ITS gene sequwence was analyzed by Unite (Release 6.0 http://unite.ut.ee/index.php)^43^.

Alpha rarefaction was performed in QIIME (version 1.7.0) using the Chao1 estimates of species abundance^44^, while observed species estimation of the amount of unique OTUs found in each sample and Shannon index^45^ were calculated. Cluster analysis was preceded by NMDS^46^. For the QIIME calculation, the beta diversity of the unweighted and weighted UniFrac distances was used for the UPGMA clustering and principal coordinate analysis^47^. To identify the differences in the bacterial communities between the two groups, similarities were analyzed using the Bray-Curtis dissimilarity distance matrices.

The metagenomic functional composition prediction analysis for all biofilm samples from 16 S data in the latest Kyoto Encyclopedia of Genes and Genomes (KEGG) database was performed using PICRUSt pipeline as described by Langille *et al*.^48^.

To associate the microbiota and flavour compounds, the significance of correlations between the microbial genera and flavour were tested(pearson correlation). The p values were adjusted by FDR using Benjamini–Hochberg method, the cutoff of adjusted p value was set as 0.05. The association network was constructed by using all significant associations, and it was displayed using R language programming.

## Electronic supplementary material

Table S1 and Figure S1
