# Supplementary material for: Microbial dynamics and metabolite changes in Chinese Rice Wine fermentation from sorghum with different tannin content
Source: Sci Rep. 2018 Mar 15;8:4639. doi: 10.1038/s41598-018-23013-1 (PMC5854674; doi:10.1038/s41598-018-23013-1)
Supplement: Supplementary file 1 — Table S1 and Figure S1 [file 41598_2018_23013_MOESM1_ESM.pdf]

Microbial dynamics and metabolite changes in Chinese Rice Wine fermentation from sorghum with different tannin content

Jialiang Xu, Huijun Wu, Zhiwei Wang, Fuping Zheng, Xin Lu, Zhenpeng Li, Qing Ren

Table. S1 Changes in acid，reducing sugar and alcohol found in JZ22(a) and JB3(b) Chinese Rice Wine brewed from Sorghum during fermentation. Every value is expressed as means ± standard error (n=3).

a

JZ22 Compounds(g/L)	Fermentation time (days)							pvalue	Correlation coefficient
	0 day	2nd day	4th day	6thday	8th day	10th day	12th day		
Acid	2.70±0.09	3.63±0.18	3.85±0.09	5.09±0.07	6.54±0.12	7.33±0.10	7.91±0.07	0.003983	-0.91
reducing sugar	18.29±0.19	19.85±0.24	17.54±0.34	17.03±0.19	16.68±0.35	15.14±0.15	14.73±0.27	2.556e-05	0.99
alcohol	2.9±0.21	5.7±0.22	8.9±0.45	9.8±0.14	10.5±0.21	10.9±0.22	11.1±0.22	0.004241	0.91

b

JB3 Compounds(g/L)	Fermentation time (days)							pvalue	Correlation coefficient
	0 day	2nd day	4th day	6thday	8th day	10th day	12th day		
Acid	1.8±0.15	2.86±0.16	3.78±0.31	4.93±0.15	5.38±0.22	6.54±0.24	7.89±0.30	0.0001436	-0.98
reducing sugar	20.64±0.23	19.25±0.16	17.61±0.14	17.23±0.19	16.67±0.21	15.72±0.25	15.15±0.12	2.259e-06	1.00
alcohol	3.1±0.08	4.3±0.14	6.5±0.22	9.2±0.17	9.6±0.22	10.4±0.08	10.8±0.14	0.0005339	0.96

Table.S2 Changes in organic acids found in JZ22(a) and JB3(b) Chinese Rice Wine during fermentation. Every value is expressed as means ± standard error (n = 3).

a

JZ22	Fermentation time (days)

Organic acid (mg/L)	0 day	2nd day	4th day	6thday	8th day	10th day	12th day
Oxalic acid	1.03±0	4.21±0.13	4.99±0.01	5.64±0.04	5.79±0.09	7.09±2.06	7.93±0.54
Tartaric acid	25.71±3.68	30.65±2.75	38.83±0.14	38.98±0.12	48.87±0.19	51.55±0.02	56.72±0.31
Pyruvic acid	0.71±0.03	1.64±0.01	22.96±0.41	22.25±0.80	15.93±0.34	15.23±0.07	14.09±0.01
Malic acid	22.06±0.20	27.17±2.09	36.85±0.01	38.02±0.28	48.70±0.63	52.41±3.06	58.86±5.31
α-ketoglutaric acid	20.59±3.12	18.44±0.24	12.03±0.29	8.60±0.13	8.27±0.08	11.72±0.34	9.92±0.28
Lactic acid	156.44±33.79	999.67±4.43	1350.52±2.29	1366.41±18.29	1449.45±5.05	1640.75±9.42	1646.58±10.33
Citric acid	3.71±0.02	17.42±0.12	18.19±0.14	19.35±0.12	19.77±0.27	22.90±0.09	26.14±0.07
Succinic acid	70.26±8.00	87.05±26.40	859.81±4.98	1236.97±2.86	1458.78±0.28	1771.71±4.71	1954.38±0.39

b

JB3	Fermentation time (days)						
Organic acid (mg/L)	0 day	2nd day	4th day	6thday	8th day	10th day	12th day
oxalic acid	1.03±0	3.32±0.01	4.52±0.12	7.79±0.06	8.52±0.02	9.37±0.16	10.68±0.15
Tartaric Acid	4.67±0.7	48.22±0.96	48.22±3.21	53.59±1.40	58.03±0.73	59.58±1.88	70.11±1.37
pyruvic acid	0.50±0.01	1.38±0.02	1.72±0.04	1.59±0.01	1.28±0.06	1.29±0.08	1.17±0.02
malic acid	24.55±0.74	32.59±1.62	46.87±0.23	48.10±0.09	50.06±8.95	58.72±0.07	62.83±2.04
α-ketoglutaric acid	1.05±0.31	8.35±0.68	6.83±0.33	5.50±0.57	4.16±0.14	5.56±0.15	5.24±0.56

lactic acid	86.69±0.89	1048.63±18.14	1351.92±36.80	1536.10±6.06	1768.18±2.971	2233.19±47.18	2084.23±7.39
citric acid	8.10±00.15	15.89±0.42	17.54±0.49	17.99±0.50	21.08±0.20	21.96±0.38	20.34±0.20
Succinic acid	150.19±1.55	182.35±0.21	634.74±5.09	687.69±0.49	863.81±14.62	1077.04±3.62	942.43±70.73

Table.S3 Changes in volatile compounds in JZ22(a) and JB3(b) Chinese Rice Wine during fermentation.

a

Relative content of volatile compounds in Jz22 fermentation stages(mg/l)								
No.	Compounds	Day 0	Day 2	Day 4	Day 6	Day 8	Day 10	Day12
alkane								
	Heneicosane	0.05		4.76	3.13	2.79		
	Pentacosane	1.50		49.86		12.68		
	Tetratriacontane	0.12		2.08	6.08			
	2,4,4-trimethyl-Hexane		0.11					
	2,6,10,15-tetramethyl-Heptadecane		0.31					
	3,7-dimethyl-Nonane	0.01	0.12	0.11	0.57		0.87	
	3,8-dimethyl-Decane	0.02						
	3-methyl-Undecane		0.18					0.50
	2,6,10-trimethyl-Dodecane	0.01						
	2,6,10,15-tetramethyl-Heptadecane				2.23			6.61
	2-ethoxy-Propane			0.58	1.69			
	Cyclodecane			0.16				
	2-Bromo dodecane					0.78		
	ethoxymethyl Oxirane				0.12			
	1,1'-Bicycloheptyl							0.08
	diethyl(decyloxy)-Borane							1.29
alcohol								
	1-Propanol		1.26	0.57	6.12	2.63	11.60	6.45
	1-Butanol		0.06		0.33	0.18	0.69	0.72

	2-methyl-1-Propanol	0.04	4.44	2.42	22.11	10.96	40.98	25.77
	1-Pentanol	0.01						
	3-methyl-1-Butanol	0.07	25.51	17.12	113.19	73.88	198.80	172.43
	1-Hexanol	0.04	0.14	0.14	0.62	0.61	1.40	1.58
	1-Heptanol		1.04					
	6-methyl-2-Heptanol							1.30
	1-Octanol	0.01		0.04		0.20	0.36	0.46
	1-Nonanol						1.05	1.54
	1-Undecanol	0.01			0.59			
	2-Furanmethanol	0.03						
	2-ethyl-2-methyl-Tridecanol		0.29					
	1-methoxy-2-Butanol						1.96	
	3-ethoxy-1-Propanol		0.05				0.77	
	2-(2-butoxyethoxy)-Ethanol		0.18					
	1-(1-methylethoxy)-2-Propanol		0.65					
	1,2-Propanediol						5.38	2.73
	Glycerin	0.03	8.36	24.27	83.56	51.26	140.53	258.38
	2,3-Butanediol	0.88	11.71	12.37	28.56	24.29	243.33	92.60
	1-Octen-3-ol	0.01						
	Phenylethyl Alcohol	0.03	0.18	7.05	14.69	40.39	105.83	93.27
	3-(methylthio)-1-Propanol					0.49	1.07	0.74
	2-Chloroethanol						4.52	
ester								
	Formic acid ethyl ester						0.42	
	Butanoic acid ethyl ester			0.10		0.25	0.30	0.36
	Pentanoic acid ethyl ester			0.08		0.19	0.40	0.51
	Hexanoic acid ethyl ester	0.01	0.19	1.74	0.91	5.73	11.32	14.17
	Heptanoic acid ethyl ester			0.11		0.58	0.77	1.16
	Octanoic acid ethyl ester		0.13	1.01	0.61	7.62	10.67	12.91
	Nonanoic acid ethyl ester			0.25	0.17	2.37	2.80	3.48

Decanoic acid ethyl ester		0.47	1.52	2.01	15.84	17.66	23.04
2-hydroxy-Propanoic acid ethyl ester		0.05	0.05	0.42	0.36	2.57	1.32
Undecanoic acid ethyl ester				0.36			
Pentadecanoic acid ethyl ester					3.80		
Dodecanoic acid ethyl ester			0.48	1.07	5.18	5.30	6.13
Tetradecanoic acid ethyl ester			0.19		2.13	2.01	1.96
Hexadecanoic acid ethyl ester	0.17		3.99	14.96	48.57	50.78	81.02
Ethyl 9-hexadecenoate					0.93		1.34
Octadecanoic acid ethyl ester					1.29		5.30
(E)-9-Octadecenoic acid ethyl ester	0.27	1.19	2.08	6.80	15.49	17.49	38.54
9,12-Octadecadienoic acid ethyl ester	0.04	0.67	1.79	6.76	10.95	12.60	29.47
Eicosanoic acid ethyl ester	0.01		0.08	0.34		1.03	
Benzoic acid ethyl ester			0.29	1.06	3.39	4.95	9.63
Benzeneacetic acid ethyl ester						0.21	
Butanedioic acid diethyl ester				0.14	0.36	1.42	2.30
Octanoic acid 3-methylbutyl ester					0.79		
3-methyl-1-Butanol acetate	0.02	0.21	0.59	0.19	0.91	2.07	2.23
Isopentyl hexanoate					0.38	0.52	
Amyl Nitrite			0.55	1.91			5.89
Acetic acid phenylethyl ester		0.12	0.06		0.42	0.90	1.23

Formic acid heptyl ester				0.23			
2-Propenoic acid oxiranylmethyl ester	0.02						
Sulfurous acid ethylhexyl hexyl ester	0.01	0.11					
Sulfurous acid ethylhexyl isohexyl ester			0.17				
Sulfurous acid butyl nonyl ester					0.56		2.30
ketone							
2-Octanone	0.01			1.49	12.32	30.58	37.18
2-Nonanone			0.06				
2-ethoxy-Propane					0.88		
1-hydroxy-2-Propanone	0.01						
1,3-dihydroxy-2-Propanone		0.60	0.13	0.54			
2-Hydroxy-gamma-butyrolactone	0.01						
3-hydroxy-2-Butanone	0.16	0.16	0.06	0.18			
4-Hydroxy-3-hexanone			0.24	0.88			
2-methyl-Cyclopentanone	0.01						
1,3-dihydroxy-2-Propanone	0.47						
dihydro-5-propyl-2(3H)-Furanone	0.01		0.04		0.61	1.23	
2,3-dihydro-3,5-dihydroxy-6-methyl-4H-Pyran-4-one	0.07		0.13				
aldehyde							
hydroxy-Acetaldehyde	0.16	2.45					
3-methyl-Hexanal			0.09				0.81
Nonanal	0.02						
Decanal	0.08						
Benzeneacetaldehyde							0.52

acid								
	Acetic acid	0.10		3.20	8.18		22.13	
	Hexanoic acid	0.16				2.00	5.04	
	2-methyl-Propanoic acid	0.07						
	3-methyl-Butanoic acid	0.14	0.57					
	hydroxy-Acetic acid						0.44	
phenol								
	Phenol	0.02					1.74	
	2-methoxy-Phenol	0.06	0.13	0.09		0.89	1.49	2.46
	4-ethyl-2-methoxy-Phenol	0.09	0.27	0.24	0.30	1.17	5.85	7.25
	2-Methoxy-4-vinylphenol	0.20	0.83	0.86	1.43	6.65	10.43	14.05
other								
	Benzothiazole	0.05	6.42	1.47	4.40	2.89	7.85	6.27
	2,3-dihydro-Benzofuran	0.01						
	2-hydroxy-N,3,3-trimethyl -Butanamide		0.07					
	2-Methoxyethyl ether		0.09					
	Acetamide		0.07					
	N-(2-methylpropyl)-Acetamide,						0.70	0.27
	Methylamine						0.13	
	Vinylfuran							1.03

b

Relative content of volatile compounds in JB3 fermentation stages(mg/l)								
No.	Compounds	Day 0	Day 2	Day 4	Day 6	Day 8	Day 10	Day12
alkane								
	Heneicosane	0.12		0.55				
	Pentacosane		2.09	0.97				
	2,4,4-trimethyl-Hexane							0.42
	3,7-dimethylnonane	0.02		0.04		0.40		0.79
	3,7-dimethyldecane							0.28

	3-methylundecane	0.03					
	3,8-dimethyundecane,	0.03					
	6-methyl- Tridecane				0.17			
	3,4,5,6-tetramethyloctane		0.05					
	2,6,10,15-tetramethyl-Heptadecane		0.40	0.17				
	cis-1-ethyl-3-methyl-Cyclopentane	0.01						
	2,2'-oxybis-Pentane	0.01						
	2-(1-hydroxy-2-propyl)-1,3-dimethyl-Cyclopentane					0.09		
	ethoxymethyl-Oxirane					0.07		
	1-(1-methylpropoxy)-Butane							0.29
	1-iodo-Decane							0.30
alcohol								
	1-Propanol		1.23	0.67	1.29	1.49	2.56	3.00
	1-Butanol		0.06	0.05	0.10	0.11	0.29	0.25
	2-methyl-1-Propanol		3.90	2.45	5.11	6.74	13.83	13.40
	1-Pentanol	0.01						
	3-methyl-1-Butanol	0.21	21.76	16.56	35.04	50.89	107.59	96.68
	1-Hexanol	0.05	0.15	0.13	0.24	0.44	1.06	0.81
	Heptanol	0.02			0.07			
	1-Octanol			0.13	0.08	0.19	0.49	0.28
	1-Nonanol					0.35		
	1-Undecanol			0.09	0.17		0.72	
	2-ethyl-1-Hexanol	0.02						
	4-methyl-3-Hexanol			0.14			0.58	0.69
	2-butyl-1-Octanol						0.35	
	6-methyl-1-Heptanol					0.17		
	2,3-dimethyl-1-Pentanol			0.01				
	3-ethoxy-1-Propanol		0.08					
	1-(1-methylethoxy)-2-Prop				0.85			

	anol							
	2-(1-methylethoxy)-1-Propanol			0.28				
	Propylene Glycol		0.38					1.67
	Glycerin	0.04	9.87	11.00	5.19	30.00	16.66	83.00
	2,3-Butanediol	3.60	23.32	10.19	13.97	8.36	30.24	53.88
	2-Dodecenal			0.06				
	2-(2-butoxyethoxy)-Ethanol	0.02						
	2-ethyl-2-methyl-Tridecanol	0.05						
	3-(methylthio)-1-Propanol,					0.41	0.73	0.37
	Phenylethyl Alcohol	0.06	7.32	5.45	7.64	35.25	85.10	76.53
ester								
	(S)-2-hydroxy-Propanoic acid ethyl ester							0.40
	Butanoic acid ethyl ester			0.09	0.19	0.28	0.49	
	Pentanoic acid ethyl ester			0.07	0.15	0.22	0.50	
	Hexanoic acid ethyl ester		0.55	1.65	2.90	6.24	12.97	1.67
	Heptanoic acid ethyl ester		0.03	0.13	0.19	0.51	1.10	
	Octanoic acid ethyl ester		0.48	0.83	1.22	4.57	9.90	1.45
	Nonanoic acid ethyl ester		0.16	0.27	0.28	1.10	2.62	0.43
	Decanoic acid ethyl ester		0.93	0.12	1.19	5.54	13.67	3.50
	2-hydroxy-Propanoic acid ethyl ester		0.04	0.04	0.08	0.12	0.21	
	Undecanoic acid ethyl ester	0.02	0.08			0.21		1.02
	Benzoic acid ethyl ester		0.11	0.18	0.55	2.03	3.95	2.42
	9-oxo-Nonanoic acid ethyl ester	0.03						
	Dodecanoic acid ethyl ester		0.26		0.25	1.20	3.23	
	Tetradecanoic acid ethyl						0.87	

ester							
Hexadecanoic acid ethyl ester	1.95	2.86	3.71	5.02	9.87	24.11	17.12
Octadecanoic acid ethyl ester	0.03						
(E)-9-Octadecenoic acid ethyl ester	0.76	1.38	1.65	2.34	2.95	10.12	7.38
9,12-Octadecadienoic acid ethyl ester		0.71		0.74	1.57	5.83	4.81
Eicosanoic acid, ethyl ester						0.53	
2-methyl-Butanoic acid ethyl ester				0.08	0.28	0.27	
3-methyl-Butanoic acid ethyl ester				0.10			
Butanedioic acid diethyl ester				0.08	0.29	1.22	1.21
3-methyl-1-Butanol acetate		0.34	0.38	0.59	0.90	2.00	
Acetic acid methylpropyl ester				0.07			
Acetic acid phenylethyl ester		0.09	0.09	0.06	0.39	0.98	0.60
Isopentyl hexanoate					0.24		
Amyl Nitrite					1.38	2.36	2.34
Formic acid heptyl ester							0.49
Sulfurous acid butyl decyl ester		0.12	0.14			1.00	
Sulfurous acid hexyl octyl ester	0.01						
Sulfurous acid ethylhexyl isohexyl ester				0.17			
Oxalic acid-6-ethyloct-3-yl heptyl ester				0.52			
Pentafluoropropionic acid		0.07					

undecenyl ester							
1,2-Benzenedicarboxylic acid bis(2-methylpropyl) ester		0.07			3.08		
aldehyde							
Nonanal	0.02		0.23		0.24	0.48	
2-methyl-Pentanal			0.06				
3-methyl-Hexanal			0.05			0.49	
Hydroxy Acetaldehyde	0.03						
Methoxy Acetaldehyde	0.03						
4-methyl-Benzaldehyde			0.07		0.28		0.54
ketone							
2-Octanone		1.14		1.18	12.42	29.78	6.56
3-hydroxy-2-Butanone	0.56	0.14	0.09	0.08			
5-hydroxy-2-Pentanone						0.48	
4-Hydroxy-3-hexanone		0.19					
1,3-dihydroxy-2-Propanone	0.04						
3,5-dimethyl-4H-Pyran-4-one		0.01					
5-ethyldihydro-2(3H)-Furanone	0.01	0.06					
5-hexyldihydro-2(3H)-Furanone						1.49	
dihydro-5-propyl-2(3H)-Furanone			0.09	0.08	0.61		1.29
acid							
Acetic acid		3.47	1.45	2.72			
Hexanoic acid	0.1		0.51				
2-methyl-Propanoic acid	0.08	0.64			0.37		
3-methyl- Butanoic acid		0.77					
phenol							
Phenol	0.04						

	2-methoxy-Phenol	0.04					0.66	
	4-ethyl-2-methoxy-Phenol	0.53	1.22	0.49	1.16	6.01	15.12	10.11
	2-Methoxy-4-vinylphenol	1.11	2.36	1.87	2.26	10.20	27.61	22.35
other								
	2,3-dihydro- Benzofuran	0.07					0.88	
	methoxy-phenyl-Oxime	0.02						
	Benzothiazole	0.17	4.96	0.97	7.40	1.74	7.36	3.75
	2-(2-butoxyethoxy)-Ethanol		0.08					
	Acetamide			0.04				
	(E)-5-Decene				0.09			
	N-(2-methylpropyl)-Acetamide					0.11	0.82	0.23
	Phenanthrene					2.55		
	1,3,5,7-Cyclooctatetraene						0.31	
	9-methylene-9H-Fluorene							2.22

Table S4. Comparisons of alpha diversity indice among the two kinds of sorghum.

Estimators	shannon	simpson
Pvalue(JB3_D0-JB3_D10)	0.008879	0.104
Pvalue(JB3_D0-JB3_D12)	0.01504	0.009197
Pvalue(JB3_D0-JB3_D2)	0.1906	0.5166
Pvalue(JB3_D0-JB3_D4)	0.0008832	0.005293
Pvalue(JB3_D0-JB3_D6)	0.003188	0.001154
Pvalue(JB3_D0-JB3_D8)	0.001705	0.000384
Pvalue(JB3_D0-JZ22_D0)	0.1258	0.2563
Pvalue(JB3_D0-JZ22_D10)	0.04263	0.03221
Pvalue(JB3_D0-JZ22_D12)	0.01823	0.007066
Pvalue(JB3_D0-JZ22_D2)	0.9167	0.363
Pvalue(JB3_D0-JZ22_D4)	0.001686	0.001699
Pvalue(JB3_D0-JZ22_D6)	0.001731	0.002269
Pvalue(JB3_D0-JZ22_D8)	0.02023	0.001444
Pvalue(JB3_D10-JB3_D12)	0.8957	0.6109

Pvalue(JB3_D10-JB3_D2)	0.9931	0.8698
Pvalue(JB3_D10-JB3_D4)	0.01753	0.06244
Pvalue(JB3_D10-JB3_D6)	0.4479	0.2676
Pvalue(JB3_D10-JB3_D8)	0.04505	0.08367
Pvalue(JB3_D10-JZ22_D0)	0.2831	0.6782
Pvalue(JB3_D10-JZ22_D10)	0.106	0.3669
Pvalue(JB3_D10-JZ22_D12)	0.7653	0.6041
Pvalue(JB3_D10-JZ22_D2)	0.1218	0.1436
Pvalue(JB3_D10-JZ22_D4)	0.08265	0.1586
Pvalue(JB3_D10-JZ22_D6)	0.05594	0.1226
Pvalue(JB3_D10-JZ22_D8)	0.6279	0.5643
Pvalue(JB3_D12-JB3_D2)	0.9752	0.7219
Pvalue(JB3_D12-JB3_D4)	0.004092	0.06039
Pvalue(JB3_D12-JB3_D6)	0.1916	0.1352
Pvalue(JB3_D12-JB3_D8)	0.006498	0.02438
Pvalue(JB3_D12-JZ22_D0)	0.2942	0.4054
Pvalue(JB3_D12-JZ22_D10)	0.001308	0.002487
Pvalue(JB3_D12-JZ22_D12)	0.4448	0.8701
Pvalue(JB3_D12-JZ22_D2)	0.1403	0.1328
Pvalue(JB3_D12-JZ22_D4)	0.02186	0.08225
Pvalue(JB3_D12-JZ22_D6)	0.009841	0.07162
Pvalue(JB3_D12-JZ22_D8)	0.1458	0.7632
Pvalue(JB3_D2-JB3_D4)	0.2435	0.3012
Pvalue(JB3_D2-JB3_D6)	0.7859	0.5374
Pvalue(JB3_D2-JB3_D8)	0.3682	0.3329
Pvalue(JB3_D2-JZ22_D0)	0.5595	0.9541
Pvalue(JB3_D2-JZ22_D10)	0.5048	0.8416
Pvalue(JB3_D2-JZ22_D12)	0.9344	0.719
Pvalue(JB3_D2-JZ22_D2)	0.2252	0.244
Pvalue(JB3_D2-JZ22_D4)	0.4739	0.4411
Pvalue(JB3_D2-JZ22_D6)	0.4057	0.3989
Pvalue(JB3_D2-JZ22_D8)	0.8871	0.7006
Pvalue(JB3_D4-JB3_D6)	0.005878	0.1082

Pvalue(JB3_D4-JB3_D8)	0.08525	0.723
Pvalue(JB3_D4-JZ22_D0)	0.03221	0.0672
Pvalue(JB3_D4-JZ22_D10)	0.001032	0.0216
Pvalue(JB3_D4-JZ22_D12)	0.00591	0.05959
Pvalue(JB3_D4-JZ22_D2)	0.0397	0.06368
Pvalue(JB3_D4-JZ22_D4)	0.02977	0.2431
Pvalue(JB3_D4-JZ22_D6)	0.0524	0.3782
Pvalue(JB3_D4-JZ22_D8)	0.006079	0.049
Pvalue(JB3_D6-JB3_D8)	0.02472	0.05254
Pvalue(JB3_D6-JZ22_D0)	0.1591	0.2133
Pvalue(JB3_D6-JZ22_D10)	0.01594	0.01471
Pvalue(JB3_D6-JZ22_D12)	0.1536	0.1342
Pvalue(JB3_D6-JZ22_D2)	0.09773	0.103
Pvalue(JB3_D6-JZ22_D4)	0.0851	0.3765
Pvalue(JB3_D6-JZ22_D6)	0.03791	0.2368
Pvalue(JB3_D6-JZ22_D8)	0.1202	0.1629
Pvalue(JB3_D8-JZ22_D0)	0.05376	0.08993
Pvalue(JB3_D8-JZ22_D10)	0.00114	0.005739
Pvalue(JB3_D8-JZ22_D12)	0.009003	0.02177
Pvalue(JB3_D8-JZ22_D2)	0.05416	0.07402
Pvalue(JB3_D8-JZ22_D4)	0.2741	0.2267
Pvalue(JB3_D8-JZ22_D6)	0.625	0.4378
Pvalue(JB3_D8-JZ22_D8)	0.009011	0.01034
Pvalue(JZ22_D0-JZ22_D10)	0.8961	0.7635
Pvalue(JZ22_D0-JZ22_D12)	0.3261	0.4016
Pvalue(JZ22_D0-JZ22_D2)	0.3042	0.1825
Pvalue(JZ22_D0-JZ22_D4)	0.071	0.1432
Pvalue(JZ22_D0-JZ22_D6)	0.05979	0.1173
Pvalue(JZ22_D0-JZ22_D8)	0.3681	0.3785
Pvalue(JZ22_D10-JZ22_D12)	0.003283	0.0001959
Pvalue(JZ22_D10-JZ22_D2)	0.3104	0.2184
Pvalue(JZ22_D10-JZ22_D4)	0.00384	0.01625
Pvalue(JZ22_D10-JZ22_D6)	0.001708	0.01737

Pvalue(JZ22_D10-JZ22_D8)	0.005956	0.01487
Pvalue(JZ22_D12-JZ22_D2)	0.1484	0.1323
Pvalue(JZ22_D12-JZ22_D4)	0.02384	0.08049
Pvalue(JZ22_D12-JZ22_D6)	0.0125	0.07012
Pvalue(JZ22_D12-JZ22_D8)	0.2049	0.7963
Pvalue(JZ22_D2-JZ22_D4)	0.06363	0.08807
Pvalue(JZ22_D2-JZ22_D6)	0.05772	0.08119
Pvalue(JZ22_D2-JZ22_D8)	0.1582	0.1279
Pvalue(JZ22_D4-JZ22_D6)	0.489	0.6832
Pvalue(JZ22_D4-JZ22_D8)	0.02202	0.07227
Pvalue(JZ22_D6-JZ22_D8)	0.01215	0.05853

Table S5. Bacterial genera abundance during different fermentation stages of JZ22 and JB3.

OTU ID	JZ22 D0	JZ22 D2	JZ22 D4	JZ22 D6	JZ22 D8	JZ22 D10	JZ22 D12	JB3 D0	JB3 D2	JB3 D4	JB3 D6	JB3 D8	JB3 D10	JB3 D12
g__[Eubacterium]_coprostanoligenes_group	0	0	0	0	0	0	0	0	0	0	2	1	0	0
g__[Eubacterium]_eligens_group	0	0	0	0	0	1	0	0	0	1	0	0	0	1
g__[Eubacterium]_hallii_group	1	0	0	0	1	1	0	0	0	1	1	2	0	2
g__[Eubacterium]_rectale_group	0	0	2	1	1	5	6	0	0	5	1	3	4	9
g__[Eubacterium]_ruminantium_group	0	0	0	0	0	0	1	0	0	0	0	0	0	0
g__[Eubacterium]_ventriosum_group	0	0	0	0	0	0	0	0	0	0	0	2	0	0
g__[Ruminococcus]_torques_group	0	0	0	0	1	1	2	0	0	0	0	5	3	1
g__Acetobacter	16	4	11	10	5	1	5	2	15	3	4	8	3	3
g__Achromobacter	1	0	3	4	4	7	3	0	1	7	5	7	5	7
g__Acidibacter	0	0	0	0	0	0	0	0	0	1	0	0	0	0
g__Acinetobacter	4	1	17	6	6	10	68	5	4	11	7	15	9	9
g__Alistipes	0	0	0	0	0	0	0	0	0	0	1	0	1	0
g__Alloprevotella	0	0	0	0	0	0	0	0	0	1	0	0	0	0
g__Anaerococcus	0	0	0	0	0	0	0	0	0	1	0	0	0	1
g__Anaerostipes	0	0	0	0	1	1	3	1	0	3	1	2	1	3
g__Apibacter	15	19	49	91	14	11	4	7	42	64	31	37	10	7
g__Aquabacterium	3	1	7	4	5	7	7	1	2	10	5	8	8	7

g__Atopobium	0	0	0	0	0	0	22	0	0	0	0	0	0	0
g__Azospirillum	0	0	1	0	0	0	0	0	0	0	0	0	0	0
g__Bacillus	37925	36148	33308	34736	37303	37942	37252	38918	33378	32132	33412	31541	35042	35857
g__Bacteroides	3	1	1	3	4	4	5	1	5	3	2	3	3	4
g__Bdellovibrio	0	0	0	1	1	1	0	0	1	0	1	1	2	3
g__Bifidobacterium	0	2	1	3	3	8	11	0	3	15	5	13	10	17
g__Bilophila	0	0	0	0	0	0	0	0	0	0	0	0	0	0
g__Blautia	1	1	2	2	3	8	11	1	2	9	4	16	8	14
g__Bradyrhizobium	0	0	1	1	1	1	2	1	1	2	1	2	1	1
g__Brevibacillus	1	0	1	88	32	7	1	38	78	972	3488	4400	1936	2080
g__Brevundimonas	1	1	3	1	0	0	0	0	1	2	0	1	0	0
g__Burkholderia-Paraburkholderia	1	1	13	18	12	15	9	2	4	7	10	17	9	12
g__Carnimonas	1	0	0	1	0	0	0	1	0	0	1	0	0	0
g__Catenibacterium	0	0	0	0	0	0	0	0	0	0	0	0	1	0
g__Cellulosimicrobium	0	0	0	0	0	0	0	0	0	0	0	0	0	1
g__Cetobacterium	0	0	0	0	0	0	0	0	0	1	0	0	1	0
g__Chroococcidiopsis	0	0	0	0	11	0	0	0	0	0	0	0	0	0
g__Chryseobacterium	0	0	0	0	0	1	9	0	0	0	0	0	0	0
g__Cloacibacterium	0	0	0	0	0	0	2	0	0	1	0	0	0	0
g__Clostridium_sensu_stricto_1	0	1	1	1	1	1	0	0	0	7	2	2	4	2
g__Clostridium_sensu_stricto_10	0	0	0	0	0	0	0	0	0	0	1	0	0	0
g__Clostridium_sensu_stricto_13	0	0	0	0	0	0	0	0	0	0	0	0	0	0
g__Collinsella	0	1	1	0	1	5	6	0	1	2	2	7	2	5
g__Coprococcus_1	0	0	0	0	0	0	0	0	0	1	0	1	0	1
g__Coprococcus_3	1	0	0	0	1	1	1	0	0	0	0	1	0	1
g__Corynebacterium_1	1	2	7	4	4	3	5	1	1	4	6	4	2	5
g__Cupriavidus	0	0	0	0	0	1	0	0	0	2	0	2	1	1
g__Deinococcus	0	0	0	0	3	0	0	0	0	0	0	0	1	0
g__Desulfovibrio	1	1	0	0	0	0	0	0	0	1	0	0	0	0
g__Dialister	0	0	0	1	0	0	4	0	0	1	1	0	0	2
g__Dorea	0	1	0	1	1	2	1	0	0	2	1	3	1	0
g__Enhydrobacter	0	0	0	1	0	3	0	0	0	0	0	0	0	5
g__Enterococcus	1	1	3	1	0	0	3	0	1	0	1	0	1	1

g__Erysipelotrichaceae_UCG-003	0	0	0	0	0	0	0	0	0	0	0	1	0	0
g__Escherichia-Shigella	2	1	3	5	4	4	8	2	3	9	7	6	3	5
g__Faecalibacterium	2	1	2	2	3	10	12	1	2	10	2	8	11	10
g__Finegoldia	0	0	0	0	1	0	0	0	0	0	0	0	0	0
g__Flavobacterium	0	0	0	0	0	0	0	0	0	0	0	1	0	0
g__Fusicatenibacter	0	0	0	0	1	2	1	1	1	3	2	8	6	4
g__Gardnerella	0	0	0	0	0	0	38	0	0	0	0	0	0	0
g__Georgenia	0	0	0	0	0	0	0	0	3	0	0	0	0	0
g__Halomonas	0	1	0	1	0	0	0	0	0	1	0	0	0	1
g__Klebsiella	10	3	21	20	13	9	75	4	15	15	10	15	13	17
g__Kroppenstedtia	14	20	123	178	102	129	93	8	60	194	134	231	177	172
g__Lachnoclostridium	0	0	0	0	0	0	0	0	0	2	0	2	2	0
g__Lachnospira	0	0	0	0	0	2	0	0	0	0	0	1	1	1
g__Lachnospiraceae_ND3007_group	0	0	0	1	0	0	0	0	0	0	0	2	1	1
g__Lachnospiraceae_NK4A136_group	0	0	1	0	0	2	3	0	1	3	0	3	1	1
g__Lachnospiraceae_UCG-004	0	0	0	0	0	0	0	0	0	1	0	0	0	0
g__Lactobacillus	42	29	60	72	19	9	11	25	26	15	11	12	24	6
g__Lactococcus	193	10	25	19	15	15	7	140	9	7	2	1	3	1
g__Lentibacillus	0	0	0	3	1	0	0	1	0	0	0	0	0	0
g__Leuconostoc	1	10	12	6	7	3	0	0	6	11	3	4	3	2
g__Lysinibacillus	0	0	0	0	0	0	0	0	0	1	1	0	1	1
g__Marmoricola	0	0	0	0	3	0	0	0	0	0	0	0	0	0
g__Megamonas	0	0	0	1	3	1	1	0	1	0	0	1	1	0
g__Megasphaera	0	0	0	0	0	0	19	0	0	0	0	0	0	0
g__Mesorhizobium	0	0	0	1	0	0	0	0	0	0	0	0	0	0
g__Methylobacterium	0	0	0	1	0	0	0	1	0	0	0	0	1	1
g__Microbispora	0	0	0	1	0	0	1	0	0	0	0	0	0	0
g__Moraxella	0	0	0	0	0	0	0	0	0	3	0	0	0	0
g__Nafulsella	0	1	0	0	0	1	0	0	0	0	0	0	0	1
g__Nitrospira	0	0	0	0	0	0	0	0	0	0	0	0	0	1
g__norank_c__1-20	0	0	0	0	0	0	0	0	3	0	0	0	0	0
g__norank_c__Acidobacteria	1	0	0	0	1	1	1	0	0	1	0	3	1	3
g__norank_c__Actinobacteria	0	1	0	0	0	0	0	0	0	0	0	0	0	0

g__norank_c__BD7-11	0	0	1	1	3	2	3	0	0	2	2	3	2	2
g__norank_c__Cyanobacteria	357	753	1042	924	674	344	272	132	2378	2728	1331	1173	1096	695
g__norank_c__Gemmatimonadetes	0	2	0	0	0	1	0	0	0	0	0	0	0	0
g__norank_f__0319-6G20	0	0	1	1	1	2	1	0	1	0	1	2	2	2
g__norank_f__Actinopolysporaceae	0	1	8	3	2	5	5	0	1	2	4	7	4	6
g__norank_f__Anaerolineaceae	0	0	0	0	0	0	0	0	0	0	1	0	0	0
g__norank_f__Bacteroidales_S24-7_group	2	0	2	0	0	0	0	0	1	2	1	0	0	1
g__norank_f__Cytophagaceae	0	0	0	0	0	0	1	0	0	0	0	0	0	0
g__norank_f__Elev-16S-1332	0	0	0	0	0	0	0	1	0	0	0	0	0	0
g__norank_f__env.OPS_17	0	0	0	0	0	0	0	0	0	0	0	0	1	0
g__norank_f__Holophagaceae	0	0	0	0	1	0	0	0	0	0	1	0	0	1
g__norank_f__Longimicrobiaceae	0	1	0	0	0	0	0	0	0	0	0	0	0	0
g__norank_f__Mitochondria	102	256	285	353	205	42	19	24	684	290	133	181	224	59
g__norank_f__Neisseriaceae	0	0	1	0	0	0	0	0	0	0	1	1	0	0
g__norank_f__Nitrosomonadaceae	0	0	0	0	1	0	0	0	0	0	0	0	0	0
g__norank_f__Porphyromonadaceae	0	0	0	1	0	0	0	0	0	0	0	0	0	0
g__norank_f__Ruminococcaceae	0	0	0	0	0	0	0	0	0	0	1	1	1	1
g__norank_f__Synergistaceae	0	0	0	0	0	0	1	0	0	0	0	0	0	0
g__norank_f__Syntrophaceae	0	0	0	0	0	0	0	0	0	0	0	1	0	1
g__norank_f__Unknown_Family	0	0	1	1	0	0	0	0	0	0	0	0	0	0
g__norank_p__Saccharibacteria	0	0	0	0	0	0	0	0	0	0	0	1	0	0
g__norank_p__WWE3	1	0	3	0	0	0	0	0	16	1	0	0	0	0
g__Novosphingobium	0	0	1	0	0	1	0	1	1	1	0	1	1	1
g__Oceanobacillus	0	3	1	1	1	0	0	0	0	1	0	4	0	0
g__Paenibacillus	940	322	180	247	41	264	898	526	1111	553	189	635	349	162
g__Paeniclostridium	1	0	0	1	0	2	1	0	0	0	1	0	1	0
g__Pantoea	1	0	4	3	2	1	0	0	8	1	1	1	2	1
g__Parabacteroides	0	0	0	0	0	0	1	0	0	0	0	1	0	0
g__Paracoccus	1	0	2	1	0	1	0	0	0	0	0	1	0	1
g__Parasutterella	1	0	0	0	0	0	0	0	1	0	0	0	0	0
g__Pediococcus	38	58	92	182	20	18	21	20	31	22	2	8	7	6
g__Pelomonas	3	1	1	6	6	7	3	2	2	14	4	6	6	9
g__Perlucidibaca	0	0	0	0	0	1	0	0	0	1	0	0	0	0

g__Phascolarctobacterium	0	0	0	0	0	0	2	0	0	1	0	0	0	0
g__Porphyromonas	0	0	0	0	0	0	2	0	0	0	0	0	0	0
g__Prevotella	0	0	0	0	0	0	70	0	0	2	0	0	0	0
g__Prevotella_1	0	0	2	0	0	0	0	0	0	0	0	0	0	0
g__Prevotella_6	0	0	0	0	0	0	5	0	0	0	0	0	0	0
g__Prevotella_9	0	3	1	3	5	18	16	2	1	2	0	3	1	2
g__Propionibacterium	0	1	2	4	2	1	3	0	0	2	1	1	1	4
g__Pseudomonas	2	4	17	7	2	4	3	0	3	7	2	4	7	2
g__Pseudorhodoferax	0	0	0	1	0	0	0	0	0	0	0	0	0	0
g__Ralstonia	43	16	114	133	108	162	128	29	38	194	90	139	144	150
g__Rhizobium	0	0	0	0	0	0	1	0	0	0	0	0	1	0
g__Rhizomicrobium	0	0	0	0	0	1	0	0	0	0	0	0	0	1
g__Rhodococcus	38	13	93	128	81	123	127	35	29	199	78	140	81	163
g__Rikenellaceae_RC9_gut_group	0	0	0	0	1	0	0	0	0	1	0	0	0	0
g__Roseburia	0	0	1	0	0	1	4	0	1	0	0	1	1	1
g__Roseomonas	0	0	0	0	0	0	0	0	0	1	0	0	0	0
g__Rubellimicrobium	0	0	2	0	0	0	0	0	0	1	0	0	0	0
g__Ruminiclostridium	0	0	0	0	0	0	0	0	0	1	0	0	0	0
g__Ruminococcaceae_UCG-002	0	0	0	0	0	0	0	0	0	0	0	0	0	1
g__Ruminococcaceae_UCG-010	0	0	0	0	0	0	0	0	0	0	0	0	0	0
g__Ruminococcus_1	0	0	1	0	0	4	1	0	0	1	0	4	2	2
g__Ruminococcus_2	2	0	0	0	0	0	1	1	0	5	0	3	1	1
g__Rummeliibacillus	6	28	5	6	2	6	5	8	44	65	31	75	25	10
g__Saccharopolyspora	7	26	82	76	47	46	41	4	34	43	58	86	47	72
g__Sediminibacterium	0	0	0	0	0	0	0	0	1	0	0	0	0	0
g__Shuttleworthia	1	1	0	0	0	0	232	0	0	1	0	0	0	0
g__Sneathia	0	0	0	0	0	0	11	0	0	0	0	0	0	0
g__Solibacillus	0	0	0	0	0	2	0	0	0	0	0	0	1	1
g__Sorangium	0	0	1	0	0	0	0	0	1	1	0	0	1	0
g__Sphingobacterium	0	2	3	3	0	0	0	0	0	1	0	1	0	0
g__Sphingomonas	0	0	0	0	1	1	6	0	0	1	0	1	1	0
g__Sporosarcina	0	0	0	0	0	0	0	0	0	1	0	0	0	0
g__Staphylococcus	8	38	86	123	22	16	11	6	76	15	7	8	5	5

g__Stenotrophomonas	0	0	1	0	1	0	6	0	0	1	0	1	3	3
g__Streptococcus	1	0	0	3	0	0	10	0	1	4	0	1	0	4
g__Streptomyces	13	79	186	206	88	119	68	3	147	102	115	132	122	144
g__Subdoligranulum	0	0	0	0	0	1	2	0	1	5	3	6	4	6
g__Sulfuritalea	0	0	0	0	0	0	0	0	0	0	0	0	0	0
g__Sva0081_sediment_group	0	0	0	0	0	0	0	1	0	0	0	0	0	0
g__Terrabacter	0	0	1	0	0	4	1	0	0	1	0	0	0	2
g__Terrisporobacter	0	0	0	0	0	0	0	0	0	1	0	0	0	0
g__Thermoactinomyces	0	0	1	0	0	1	0	0	0	1	2	2	1	0
g__Thermus	0	0	0	0	0	0	1	0	0	1	0	1	1	0
g__unclassified_c__Betaproteobacteria	0	0	0	0	0	1	0	0	0	0	0	0	1	0
g__unclassified_c__Gemmatimonadetes	0	0	1	0	0	0	0	0	0	0	0	0	0	0
g__unclassified_f__Comamonadaceae	0	0	0	0	3	0	0	0	0	0	0	0	0	0
g__unclassified_f__Corynebacteriaceae	0	0	1	1	0	0	1	0	0	1	0	0	1	0
g__unclassified_f__Enterobacteriaceae	1	1	4	6	4	0	0	0	0	4	8	3	4	0
g__unclassified_f__Erythrobacteraceae	0	0	0	0	0	0	0	0	0	0	0	1	0	0
g__unclassified_f__Lachnospiraceae	0	0	0	0	0	0	2	0	1	1	0	2	1	0
g__unclassified_f__Peptostreptococcaceae	0	0	0	1	1	1	1	0	1	2	1	1	3	2
g__unclassified_f__Prevotellaceae	0	0	0	1	0	0	1	0	0	0	0	0	0	0
g__unclassified_k__norank	0	2	4	0	0	0	1	1	4	5	2	1	9	1
g__unclassified_o__Bacillales	0	0	0	1	0	0	0	1	2	3	12	28	12	6
g__unclassified_o__Rhizobiales	0	0	0	0	1	0	0	0	0	0	0	1	0	0
g__unclassified_p__Firmicutes	0	0	0	0	0	0	0	0	0	0	0	0	0	1
g__unclassified_p__Proteobacteria	10	4	26	3	6	8	1	1	19	13	16	6	21	8
g__Undibacterium	0	0	0	0	0	1	0	0	0	1	1	2	1	1
g__Vibrio	0	0	0	0	1	0	0	0	0	1	0	0	0	0
g__Virgibacillus	0	0	0	0	0	0	0	0	0	1	0	0	0	0
g__Weissella	192	2134	4073	2294	1087	564	312	51	1702	2154	743	933	495	187

Table S6. Predicted gene functions related to KEGG pathways at levels 3

	JZ22 D0	JZ22 D2	JZ22 D4	JZ22 D6	JZ22 D8	JZ22 D10	JZ22 D12	JB3 D0	JB3 D2	JB3 D4	JB3 D6	JB3 D8	JB3 D10	JB3 D12
1,1,1-Trichloro-2,2-bis(4-chlorophenyl)ethane	34	13	89	113	83	128	112	28	30	173	74	121	100	137

(DDT) degradation														
ABC transporters	679287	652100	678970	679262	685263	681308	716107	667532	726606	730771	710166	712650	703137	697452
Adherens junction	0	0	0	0	0	0	0	0	0	0	0	0	0	0
Adipocytokine signaling pathway	10320	9943	10688	10885	10831	10801	10901	10001	11204	11864	10842	10808	10882	11010
African trypanosomiasis	123	259	315	389	235	80	86	38	697	422	442	592	413	286
Alanine, aspartate and glutamate metabolism	174464	170413	170436	173142	177555	178298	182199	176133	186216	184978	180232	176859	180330	179939
Aldosterone-regulated sodium reabsorption	0	0	0	0	0	0	0	0	0	0	0	1	1	0
Alzheimer's disease	10367	11059	11284	11665	10892	10256	10284	10001	13547	11672	10522	10517	10917	10184
Amino acid metabolism	56996	56797	57012	57168	59015	58794	57525	58572	62986	63327	62583	61926	61687	61042
Amino acid related enzymes	223291	226579	231517	231650	229869	226134	232042	223180	256962	249503	232257	229863	233594	227814
Amino sugar and nucleotide sugar metabolism	239585	234376	237317	236608	241945	243190	250156	243180	258577	250236	242842	236757	243631	241472
Aminoacyl-tRNA biosynthesis	154756	164350	174653	171099	163322	156448	161215	151395	193473	188449	165086	165581	166218	158478
Aminobenzoate degradation	89724	85786	85733	87968	91638	93950	92263	93412	89300	91345	92258	92029	93290	95501
Amoebiasis	13290	12536	11807	12111	13347	13725	13333	14052	13125	12009	12818	12185	13249	13317
Amyotrophic lateral sclerosis (ALS)	22104	20794	18887	19800	22013	22862	22112	23853	21242	19119	20988	20552	22201	22382
Antigen processing and presentation	5080	5085	4868	5114	5009	4967	5216	4904	6113	5560	5140	4918	5081	4919
Apoptosis	170	697	1159	884	458	208	143	51	1040	778	302	415	366	148
Arachidonic acid metabolism	14484	14159	13441	14019	14751	14891	14497	14701	15180	14992	14810	14580	15102	15029
Arginine and proline metabolism	242204	236274	240807	242393	247131	245820	251878	241568	261597	261500	249952	246353	250341	248340
Arrhythmogenic right ventricular cardiomyopathy (ARVC)	0	0	0	0	0	0	0	0	0	0	0	0	0	0
Ascorbate and aldarate metabolism	54560	50349	49574	50534	54361	56019	56159	56564	51955	47896	51468	49117	52765	53853
Atrazine degradation	2656	3115	4442	4407	2847	2130	2769	892	4641	6058	2911	2736	2365	2166
Bacterial chemotaxis	128790	119861	113136	117396	125367	128817	137506	131072	118220	113501	124581	123079	125731	128063
Bacterial invasion of epithelial cells	181	366	512	448	341	170	145	134	1237	1534	750	678	609	408
Bacterial motility proteins	27587	26438	25472	26296	27546	27703	29198	27523	27900	27806	27943	27540	28019	28001

	2	1	0	6	3	1	2	1	5	2	8	1	6	1
Bacterial secretion system	73197	78532	82884	82780	77584	73420	75031	70416	95529	88801	76689	76368	78023	73334
Bacterial toxins	14623	14618	15354	14977	15118	14993	15081	15248	16160	17367	16550	16737	16146	15990
Basal transcription factors	116	57	115	149	75	116	162	66	176	253	389	553	288	305
Base excision repair	66617	67500	69144	69848	68625	67498	68996	65384	75688	75121	70358	71073	70534	69183
Benzoate degradation	71340	69913	73739	75200	73864	75500	74986	71770	70997	79454	76041	78338	75682	78382
Betalain biosynthesis	12	18	57	65	41	58	40	7	34	64	40	56	55	61
Bile secretion	7	29	77	81	36	47	39	2	55	42	46	58	51	59
Biosynthesis and biodegradation of secondary metabolites	9751	10161	10293	10542	10220	10436	10193	9977	11442	12167	11153	11047	10816	10928
Biosynthesis of 12-, 14- and 16-membered macrolides	15	85	217	231	107	136	83	2	156	116	133	164	139	170
Biosynthesis of ansamycins	13598	12706	12155	12498	13559	14072	14898	14301	13609	12650	13152	12802	13666	13786
Biosynthesis of siderophore group nonribosomal peptides	29198	28082	24021	25701	28763	30823	28755	33226	29093	27265	29330	27475	29834	30564
Biosynthesis of type II polyketide backbone	15	75	205	218	95	125	85	5	140	110	125	165	135	165
Biosynthesis of type II polyketide products	4	17	55	55	25	32	24	2	33	28	33	47	35	44
Biosynthesis of unsaturated fatty acids	56356	54543	52742	54638	57104	58528	57628	58258	58960	57366	57327	56294	58297	58706
Biosynthesis of vancomycin group antibiotics	885	1342	2587	1969	1353	863	1589	360	2999	3725	2023	2164	1668	1258
Biotin metabolism	28214	27191	26961	27491	28632	28798	29648	28894	31060	30171	29315	27939	29092	28905
Bisphenol degradation	39900	37674	36312	37550	40243	42326	41123	42836	38514	36669	38741	37664	40040	41446
Bladder cancer	23	23	94	107	68	94	86	16	49	113	66	99	80	108
	15754	15452	15817	16129	16178	16353	16377	15838	16038	16392	16223	16260	16245	16558
Butanoate metabolism	9	4	2	3	8	7	0	0	5	4	6	8	1	8
Butirosin and neomycin biosynthesis	5850	5956	7082	6645	6266	5813	6796	5262	7499	7864	6579	6869	6519	6157
C5-Branched dibasic acid metabolism	48767	46900	45519	47342	48462	49266	50428	49367	52528	51526	49544	48223	49552	49524
CAM ligands	0	0	0	0	0	0	0	0	0	0	0	0	0	0
Caffeine metabolism	310	104	600	442	334	191	440	36	41	69	47	72	56	75
Calcium signaling pathway	177	365	504	442	334	168	133	65	1177	1340	662	581	538	342
Caprolactam degradation	16520	15213	18362	18810	17927	18155	18844	15261	15155	17902	16856	17907	17092	18550
Carbohydrate digestion and absorption	5075	4898	4841	4903	5196	5049	5310	5023	5532	5463	4918	4836	5197	5014
Carbohydrate metabolism	52518	49552	50084	50181	52433	52702	54131	52821	55200	52291	51515	49220	51866	51506
Carbon fixation in photosynthetic organisms	63864	67007	72598	71191	68337	64470	67857	60982	85703	86719	72013	71287	71254	67043

	16151	15991	16211	16598	16587	16404	16666	15931	17498	17379	16685	16692	16799	16664
Carbon fixation pathways in prokaryotes	3	8	8	9	4	3	4	2	3	6	3	1	2	3
Cardiac muscle contraction	356	770	950	1155	721	276	216	101	2070	1023	466	670	793	321
Carotenoid biosynthesis	1514	3008	4299	3846	2824	1534	1267	591	9604	10973	5440	4879	4470	2957
Cell cycle	0	0	0	0	0	0	0	0	0	0	0	0	0	0
Cell cycle - Caulobacter	71150	74239	76828	76795	74796	71835	73179	70001	89560	86435	77251	77077	77541	73769
Cell cycle - yeast	0	0	0	0	0	0	0	0	0	0	0	0	0	0
Cell division	9290	9293	8907	9267	9642	9716	9605	9675	11287	10836	9982	9624	10132	9858
Cell motility and secretion	7679	10115	12142	11628	9629	7748	8377	6178	18145	18938	12362	12749	11723	9582
Cellular antigens	10537	10333	10422	10713	10646	10468	10542	10059	11163	11704	11194	10973	10821	10810
Chagas disease (American trypanosomiasis)	117	257	311	379	230	79	87	34	689	338	150	223	251	112
	12337	13133	13874	13646	13148	12572	12876	12003	15985	15750	13521	13348	13504	12808
Chaperones and folding catalysts	5	1	3	4	5	8	0	8	2	9	1	6	6	5
Chloroalkane and chloroalkene degradation	58709	58221	59124	59648	60000	60723	59860	59545	61795	63641	59940	59130	60110	60841
Chlorocyclohexane and chlorobenzene degradation	9250	9480	9312	9934	9856	10359	9613	9869	12075	12833	11001	10633	10767	10919
Cholinergic synapse	0	0	0	0	0	0	0	0	0	0	0	0	0	0
	27107	27346	27502	27683	27724	27441	28004	27083	30693	30016	27859	27135	27951	27371
Chromosome	0	8	3	2	5	7	1	0	1	5	5	9	7	6
Chronic myeloid leukemia	0	0	0	0	0	0	0	0	0	0	0	0	0	0
Circadian rhythm - plant	4763	4464	4159	4342	4639	4757	4730	4801	4195	3895	4375	4273	4489	4594
	12614	12448	12578	12985	12890	12714	12892	12398	13509	13026	12671	12597	12825	12738
Citrate cycle (TCA cycle)	8	7	9	0	3	6	9	3	5	3	2	6	5	9
Clavulanic acid biosynthesis	0	0	0	0	2	0	0	0	0	0	0	0	0	0
Colorectal cancer	108	252	292	351	209	52	32	28	679	293	131	193	232	71
Complement and coagulation cascades	0	0	0	0	0	0	0	0	0	0	0	0	0	0
Cyanoamino acid metabolism	56074	54146	52833	53863	56609	57734	59857	57993	59785	57118	56623	55312	57726	57559
	18332	17756	17162	17789	18274	18394	18853	18368	18866	18868	18528	18245	18563	18589
Cysteine and methionine metabolism	6	0	5	3	4	1	0	1	5	3	5	8	5	7
Cytochrome P450	15	5	38	51	33	50	51	14	12	80	31	56	33	66
Cytokine receptors	0	0	0	0	0	0	0	0	0	0	0	0	0	0
Cytokine-cytokine receptor interaction	0	0	0	0	0	0	0	0	0	0	0	0	0	0
Cytoskeleton proteins	41254	43243	45042	44473	43439	41815	44124	40123	52041	51961	44565	43986	44607	42543
Cytosolic DNA-sensing pathway	0	0	0	0	0	0	0	0	0	0	0	0	0	0

D-Alanine metabolism	26997	26505	25139	25593	26990	27856	27334	28958	27707	26876	27530	26491	27560	27773
D-Arginine and D-ornithine metabolism	4677	4455	4183	4357	4643	4733	4651	4886	4228	4214	4532	4391	4584	4705
D-Glutamine and D-glutamate metabolism	25084	25316	26617	26187	25899	25293	26049	24673	28394	27702	25701	25332	25790	25168
DNA repair and recombination proteins	380583	396203	414257	410497	397655	384699	393683	372347	457803	452444	403992	403337	405174	390572
DNA replication	82750	87366	92177	90762	87056	83414	85560	80681	99647	96932	85934	86150	87244	83631
DNA replication proteins	140639	148397	157008	154642	147783	141497	146247	136391	171359	167797	147803	147931	148949	142574
Dilated cardiomyopathy (DCM)	0	0	0	0	0	0	0	0	0	0	0	0	0	0
Dioxin degradation	18133	18677	18475	18428	18718	19364	18363	19656	20326	21136	20519	20357	20036	20228
Drug metabolism - cytochrome P450	16920	18374	20328	20115	18718	17716	17340	16163	24897	27706	21084	21149	20481	19393
Drug metabolism - other enzymes	47232	46920	46021	46421	47409	48616	48774	48868	47778	47042	47413	46572	47582	48205
ECM-receptor interaction	0	0	0	0	0	0	0	0	0	0	0	0	0	0
Electron transfer carriers	8971	8650	7999	8349	8991	9307	8950	9491	9299	8821	9019	8579	9117	9207
Endocrine and other factor-regulated calcium reabsorption	0	0	0	0	0	0	0	0	0	0	0	1	1	0
Endocytosis	8	3	19	26	16	26	38	7	6	41	16	28	17	34
Energy metabolism	113345	111226	112164	114399	115925	116348	119432	113437	122334	121869	116477	115532	117455	117195
Epithelial cell signaling in Helicobacter pylori infection	10392	10247	9622	10135	10049	10211	10711	10146	11300	11524	10480	10093	10239	10233
ErbB signaling pathway	0	0	0	0	0	0	0	0	0	0	0	0	0	0
Ether lipid metabolism	51	39	178	200	130	178	171	104	131	415	214	304	218	284
Ethylbenzene degradation	16578	16203	16179	16781	16333	16348	16473	16660	16405	19623	17277	17718	17068	17359
Fat digestion and absorption	0	0	0	0	0	0	0	0	0	0	0	0	0	0
Fatty acid biosynthesis	115245	112822	113770	116082	116880	116211	117398	114059	121253	119648	117276	116793	117923	117292
Fatty acid elongation in mitochondria	0	0	0	0	0	0	0	0	0	0	0	0	0	0
Fatty acid metabolism	101500	99174	104734	106716	105969	106773	106529	100221	104892	112373	105875	107079	106148	108947
Fc epsilon RI signaling pathway	0	0	0	0	0	0	0	0	0	0	0	0	0	0
Fc gamma R-mediated phagocytosis	8	3	19	26	16	26	38	7	6	41	16	28	17	34
Flagellar assembly	15640	14588	13685	14236	15279	15686	16349	15981	13940	13305	14682	14416	15022	15364

	5	0	7	1	2	6	0	0	6	8	3	3	9	4
Flavone and flavonol biosynthesis	97	36	23	31	7	33	335	55	116	63	22	74	43	27
Flavonoid biosynthesis	5343	5577	5720	5725	5665	5297	5157	5034	7767	8030	6679	6429	6299	5833
Fluorobenzoate degradation	519	850	1589	1553	1131	970	903	258	2618	3484	1753	1837	1616	1402
Focal adhesion	0	0	0	0	0	0	0	0	0	0	0	0	0	0
Folate biosynthesis	87744	87705	87163	88101	89200	89288	88914	88724	96531	96401	90414	87700	90205	89365
	15258	14620	14940	14909	15412	15471	15916	15455	16074	15526	15251	14811	15356	15282
Fructose and mannose metabolism	4	1	4	5	4	3	0	5	0	3	6	0	3	9
	27921	27378	28371	28221	28559	28248	29045	27723	30059	29990	28746	28613	28864	28514
Function unknown	5	8	4	5	2	8	8	9	3	2	2	1	6	6
G protein-coupled receptors	0	0	0	0	0	0	0	0	0	0	0	0	0	0
GTP-binding proteins	0	0	0	0	0	0	0	0	0	0	0	0	0	0
							10426							
Galactose metabolism	97261	93254	96582	95503	97069	97794	6	96769	98112	93057	91785	89355	93659	93897
Gastric acid secretion	0	0	0	0	0	0	0	0	0	0	0	1	1	0
	65181	64527	66777	66628	66783	65986	67775	64483	72436	72429	67600	66757	67624	66550
General function prediction only	9	7	7	4	0	9	5	8	1	1	8	6	1	6
Geraniol degradation	45192	42982	44682	46636	46814	48021	47887	44568	42138	45223	44871	45392	45634	48125
								10156						
Germination	97476	90651	81482	85879	92608	96992	96483	0	88816	82519	92493	90048	93260	95366
Glioma	0	0	0	0	0	0	0	0	0	0	0	0	0	0
Glutamatergic synapse	15046	14858	15772	15641	15579	15246	15857	14707	17297	17002	15949	15770	15817	15489
Glutathione metabolism	45968	49408	51065	50815	49274	47764	46650	45651	61360	62898	52813	52076	52357	49850
Glycan bindng proteins	0	0	0	0	0	0	0	0	0	0	0	0	0	0
Glycan biosynthesis and metabolism	528	802	1241	1161	875	625	697	304	2628	3339	1891	1967	1597	1212
Glycerolipid metabolism	71369	70658	74260	73730	72504	71774	74756	69557	75730	76456	72012	71123	71507	71385
									10318	10175				
Glycerophospholipid metabolism	94150	94526	95049	96273	96125	95474	97782	94330	0	1	98065	97749	98098	96929
	14772	14345	14542	14808	15008	15027	15407	14772	15656	15488	15131	14944	15160	15149
Glycine, serine and threonine metabolism	1	5	3	8	0	6	2	4	4	7	0	9	0	6
	21576	21613	22663	22429	22207	21866	22438	21339	23755	23894	22230	21929	22189	21923
Glycolysis / Gluconeogenesis	3	3	5	8	4	2	3	5	1	0	9	7	8	2
Glycosaminoglycan biosynthesis - chondroitin	0	0	0	0	0	0	0	0	0	0	0	0	0	0

sulfate														
Glycosaminoglycan degradation	128	122	240	271	124	186	529	70	280	278	445	620	356	387
Glycosphingolipid biosynthesis - ganglio series	25	66	160	175	84	115	144	9	121	100	96	124	110	135
Glycosphingolipid biosynthesis - globo series	5411	4816	5119	5219	5125	5233	6389	5022	4815	4381	4924	5133	5010	5141
Glycosphingolipid biosynthesis - lacto and neolacto series	0	0	0	0	0	0	1	0	0	0	0	0	0	0
Glycosylphosphatidylinositol(GPI)-anchor biosynthesis	0	0	0	0	0	0	0	0	0	0	0	0	0	0
Glycosyltransferases	45483	51100	53840	52070	49124	47408	46947	45314	64906	67344	53286	51011	51608	48868
Glyoxylate and dicarboxylate metabolism	126400	122008	122596	125632	128619	129515	131929	126231	130172	127980	126636	123648	127586	128527
GnRH signaling pathway	8	3	19	26	16	26	38	7	6	41	16	28	17	34
Hedgehog signaling pathway	0	0	0	0	0	0	0	0	0	0	0	0	0	0
Hematopoietic cell lineage	0	0	0	0	0	0	0	0	0	0	0	0	0	0
Hepatitis C	0	0	0	0	0	0	0	0	0	0	0	0	0	0
Histidine metabolism	92833	90413	94870	94686	94691	93597	98312	90894	98168	102586	95349	95359	95346	94989
Homologous recombination	99688	107551	114758	112411	105954	100709	103883	96254	126742	124527	106704	106601	107132	101780
Huntington's disease	14726	14841	14394	15164	15353	14796	14404	14832	18429	16425	15642	15719	16175	15295
Hypertrophic cardiomyopathy (HCM)	1	0	1	0	1	1	3	0	0	2	0	3	3	4
Indole alkaloid biosynthesis	3	14	31	35	16	21	12	1	25	19	20	25	23	27
Influenza A	110	265	323	385	224	72	43	28	703	310	150	215	252	95
Inorganic ion transport and metabolism	24932	24470	25711	26016	25771	26157	26615	25544	28837	30280	28884	29881	28273	28241
Inositol phosphate metabolism	59653	55887	56091	56561	59868	61238	61212	61654	58673	53680	56654	54649	58291	58999
Insulin signaling pathway	6766	7396	8340	8136	7529	6539	7187	5715	11878	13252	8956	8690	8468	7459
Ion channels	5042	5735	6418	5977	5320	5164	5250	5044	6131	6502	5515	5265	5189	5106
Isoflavonoid biosynthesis	93	53	167	118	231	129	154	128	143	317	181	370	347	267
Isoquinoline alkaloid biosynthesis	5632	5344	6019	6059	5992	5658	6253	5248	6568	6674	6012	6392	6218	5985
Leishmaniasis	0	0	0	0	0	0	0	0	0	0	0	0	0	0
Leukocyte transendothelial migration	0	0	0	0	0	0	0	0	0	0	0	0	0	0
Limonene and pinene degradation	76251	71646	72386	74738	77399	79793	79098	78683	72518	74945	75768	75269	76937	79943
Linoleic acid metabolism	13932	13322	13058	13430	13926	14604	14467	14619	13048	13068	13433	13339	13743	14367

Lipid biosynthesis proteins	140327	138889	141587	144397	144235	143855	145011	138928	151753	152135	145413	144943	146050	145806
Lipid metabolism	11831	12306	14788	14384	13412	12206	13218	10595	16970	17839	14259	14836	14339	13265
Lipoic acid metabolism	15302	15856	16064	16274	15866	15292	14995	14962	19277	18853	16545	16002	16326	15602
Lipopolysaccharide biosynthesis	1760	2802	4555	4144	3373	2364	2696	788	8773	10762	5362	5373	4947	3672
Lipopolysaccharide biosynthesis proteins	7160	9077	12090	10794	9283	7994	8841	6038	15666	18395	12136	12538	11265	9758
Long-term depression	0	0	0	0	0	0	0	0	0	0	0	0	0	0
Long-term potentiation	0	0	0	0	0	0	0	0	0	0	0	0	0	0
Lysine biosynthesis	102083	102192	104987	105737	104503	102597	106499	100077	114605	113107	105880	105896	106231	104082
Lysine degradation	67351	63718	68140	69488	69321	69099	70714	64682	63683	66522	65311	65794	66393	68512
Lysosome	434	233	328	386	169	318	1118	238	628	446	246	530	351	345
MAPK signaling pathway	0	0	0	0	0	0	0	0	0	0	0	0	0	0
MAPK signaling pathway - yeast	9709	9336	8682	9062	9839	9889	9627	10272	10108	10636	10148	10309	10540	10381
Measles	0	0	0	0	0	0	0	0	0	0	0	0	0	0
Meiosis - yeast	4760	5295	5455	5448	5424	5140	4688	4869	8627	8867	6593	6049	6246	5688
Melanogenesis	9	4	26	30	25	37	28	6	9	45	20	31	32	34
Membrane and intracellular structural molecules	76070	75917	75437	76980	79219	78798	79219	78638	91254	89106	82867	81448	83611	81160
Metabolism of cofactors and vitamins	30382	29470	30113	30555	30444	30509	31224	30260	32728	34375	31979	31223	31157	31181
Metabolism of xenobiotics by cytochrome P450	16689	18296	20096	19892	18404	17456	16971	15915	24640	27068	20541	20231	19872	18811
Methane metabolism	157296	158018	162019	161888	162202	162121	167053	158098	178921	180774	165513	162750	165334	163526
Mineral absorption	5428	5628	5805	5840	5721	5393	5314	5142	7966	8372	6829	6651	6453	5981
Mismatch repair	103547	109402	115231	113513	108685	104325	107608	100617	126453	123544	109379	109317	109883	105274
N-Glycan biosynthesis	4656	4945	4853	4888	5014	4899	4575	4825	7527	7421	5881	5416	5660	5232
NOD-like receptor signaling pathway	5082	5085	4868	5129	5015	4969	5235	4978	6178	5913	5807	5744	5471	5328
Naphthalene degradation	58732	58501	58268	59503	59529	60827	59442	60688	60733	65089	60756	60063	60514	61827
Neuroactive ligand-receptor interaction	2	13	31	34	15	20	11	0	24	17	19	22	20	24
Neurotrophin signaling pathway	0	0	0	0	0	0	0	0	0	0	0	0	0	0
Nicotinate and nicotinamide metabolism	57218	58304	62168	60990	59810	58156	60621	55668	65884	68901	61535	61543	61005	59659
Nitrogen metabolism	163350	157154	152168	156130	164115	167673	168689	169299	168826	163368	164435	159661	166149	167004

Nitrotoluene degradation	956	745	2384	2065	1514	1250	2411	240	1640	2304	1251	1605	1274	1339
Non-homologous end-joining	13883	12960	12227	12824	13767	14378	14102	14438	12825	12102	13680	13755	14021	14416
Notch signaling pathway	0	0	0	0	0	0	0	0	0	0	0	0	0	0
Novobiocin biosynthesis	15587	15080	15665	15969	16146	15779	16744	15145	17519	17751	16157	16237	16489	16156
Nucleotide excision repair	55653	57227	59739	59057	57750	56489	58320	54787	64242	62664	58124	58453	58523	56984
Nucleotide metabolism	9059	8768	9435	8913	9063	9318	9882	9565	8933	8674	9511	9499	9210	9356
Olfactory transduction	0	0	0	0	0	0	0	0	0	0	0	0	0	0
One carbon pool by folate	71123	73268	76391	75810	74010	72321	74721	69955	84060	83026	74896	74580	75225	73021
Oocyte meiosis	0	0	0	0	0	0	0	0	0	0	0	0	0	0
Other glycan degradation	10733	9404	10316	10000	9713	10174	13152	10100	11315	9711	9405	9784	9777	9646
Other ion-coupled transporters	415150	409359	388859	396546	417233	427627	418972	437068	418043	397415	408150	392855	415162	418617
Other transporters	30610	31238	32406	32138	31554	30809	32179	30043	37666	37806	33258	32661	32734	31580
Other types of O-glycan biosynthesis	0	0	0	0	0	0	0	0	0	0	0	0	0	0
Others	271560	266299	265027	266778	275608	277243	277338	280385	295017	294011	284428	278476	284190	281566
Oxidative phosphorylation	179896	186282	192384	195414	190099	182079	184231	175954	231780	225814	201311	200874	200200	190641
PPAR signaling pathway	16658	16840	19240	19379	18117	17852	18167	15611	20183	22994	19216	19585	18686	18950
Pancreatic cancer	0	0	0	0	0	0	0	0	0	0	0	0	0	0
Pancreatic secretion	2	13	31	34	15	20	11	0	24	17	19	23	21	24
Pantothenate and CoA biosynthesis	90399	90196	92331	92770	92723	91705	94992	89823	103367	104515	96030	94693	95349	93582
Parkinson's disease	464	1022	1242	1506	931	328	249	129	2749	1316	597	862	1024	392
Pathogenic Escherichia coli infection	0	0	0	0	0	0	0	0	0	0	0	0	0	0
Pathways in cancer	10273	10466	10226	10755	10449	10061	10222	9938	12967	11654	10579	10321	10688	10129
Penicillin and cephalosporin biosynthesis	9573	8939	8982	9227	9519	9806	9798	9905	9933	9970	10126	10049	10004	10137
Pentose and glucuronate interconversions	156322	147487	144552	145508	153939	158571	161998	161047	149696	136137	142961	135776	147221	149410
Pentose phosphate pathway	132133	134104	137545	135522	135263	134828	137888	133255	150401	149715	136969	133719	136804	134457
Peptidases	329212	330524	332154	334767	338555	334890	339850	330449	374985	374849	352591	346065	349453	342846

Peptidoglycan biosynthesis	103509	109957	115589	113384	108177	104426	107563	101094	125669	125061	111144	111188	110292	106237
Peroxisome	52200	50810	49485	51131	53207	54013	53285	53767	55430	54319	53338	52484	54271	54414
Pertussis	236	396	671	611	470	351	383	100	1254	1574	785	761	704	528
Phagosome	0	0	0	0	0	0	0	0	0	0	0	0	0	0
Phenylalanine metabolism	28940	27089	32137	32189	31444	30197	32425	26753	32327	36755	32725	35334	33107	33001
Phenylalanine, tyrosine and tryptophan biosynthesis	106678	103097	103162	105774	108283	107572	113767	105233	117318	119280	110612	109585	111367	109890
Phenylpropanoid biosynthesis	23986	22021	22341	22440	23760	24144	27021	24185	25859	24085	23510	22983	24170	23833
Phosphatidylinositol signaling system	15708	16857	18050	17559	16662	15843	16103	15196	20260	20267	17632	17655	17306	16440
Phosphonate and phosphinate metabolism	9236	8485	7830	8340	8829	9413	9748	9895	8776	8686	9662	9762	9564	9801
Phosphotransferase system (PTS)	232014	214100	207000	210306	228326	235693	235033	243060	207955	186492	210258	198493	217169	222350
Photosynthesis	51770	66060	76996	72345	63506	51796	52498	44042	121747	129673	82938	78113	75948	62257
Photosynthesis - antenna proteins	3013	6205	8568	7518	5670	2860	2261	1109	20013	22780	11250	9881	9146	5810
Photosynthesis proteins	59752	77121	90320	84742	74204	59654	59696	50162	147290	158079	99159	92749	90161	73028
Phototransduction	0	0	0	0	0	0	0	0	0	0	0	0	0	0
Phototransduction - fly	0	0	0	0	0	0	0	0	0	0	0	0	0	0
Plant-pathogen interaction	20186	20163	20655	20752	20335	20191	21705	19761	21653	21137	21008	20939	20552	20439
Polycyclic aromatic hydrocarbon degradation	33274	33621	35638	34931	34843	35209	35136	34434	36319	37264	34620	34188	34873	35192
Polyketide sugar unit biosynthesis	3807	5540	9964	7714	5105	3319	5608	1572	11140	13682	6926	7318	5758	4287
Pores ion channels	24254	27249	29303	29234	26798	24482	25339	21902	41461	46217	33133	31946	30810	27940
Porphyrin and chlorophyll metabolism	115876	118906	130072	129389	125366	116143	122163	107500	170726	176186	143895	142771	138502	128150
Prenyltransferases	51903	54764	56741	56733	54691	52450	52558	50640	68463	68285	58440	57419	57535	54638
Primary bile acid biosynthesis	4169	4299	4353	4282	4355	4681	4805	4673	4288	4094	4155	4036	4258	4493
Primary immunodeficiency	5124	4997	5154	5344	5273	5339	5557	5055	5721	5886	5427	5415	5462	5482
Prion diseases	4286	3925	3596	3722	4429	4600	4416	4813	4165	3881	4415	4606	4788	4767
Progesterone-mediated oocyte maturation	5080	5085	4868	5114	5009	4967	5216	4904	6113	5560	5140	4918	5081	4919
Propanoate metabolism	163478	156052	160048	163922	166988	168152	170369	162557	159465	162059	163452	163881	165303	168876

Prostate cancer	5080	5085	4868	5114	5009	4967	5216	4904	6113	5560	5140	4918	5081	4919
Proteasome	5127	5173	5155	5433	5181	5205	5414	4940	6289	5840	5334	5199	5285	5229
Protein digestion and absorption	10	36	91	102	49	68	98	7	69	139	347	446	226	254
Protein export	99617	103547	104890	105387	102695	100610	101668	99118	117584	112608	102899	100781	103294	100293
Protein folding and associated processing	66562	69537	77524	76161	72812	67959	70439	62128	95083	98886	78502	78585	77731	72479
Protein kinases	154211	148867	142700	145977	153981	156713	155845	159335	164828	156915	157951	153123	158246	157215
Protein processing in endoplasmic reticulum	14337	14460	13942	14432	14706	14644	14390	14474	17657	16872	15408	14543	15220	14783
Proximal tubule bicarbonate reclamation	9582	8845	9194	9224	9669	9708	9855	9543	9364	9053	9393	9074	9413	9524
Purine metabolism	331818	341787	360738	354032	343903	335389	344555	324549	381902	381541	344379	340174	343438	335176
Pyrimidine metabolism	240968	250345	262897	258409	250138	243050	250120	236038	278746	276816	251520	251383	251732	244634
Pyruvate metabolism	235157	233372	236466	238360	241121	240315	241734	236646	252727	253369	243338	240204	243708	242451
RIG-I-like receptor signaling pathway	443	141	633	481	346	223	570	160	211	320	154	236	165	157
RNA degradation	62577	65642	69025	68380	65995	63051	65009	60606	80561	80907	69495	68530	68561	65194
RNA polymerase	25734	27573	29247	28527	27211	25981	26049	25104	31989	31197	27270	27122	27403	26103
RNA transport	20380	20920	22348	21428	20957	20455	21407	19940	22177	22529	20618	20377	20562	20154
Regulation of actin cytoskeleton	0	0	0	0	0	0	0	0	0	0	0	0	0	0
Renal cell carcinoma	5085	5129	5066	5290	5231	5042	4974	5006	6175	5801	5308	5210	5375	5139
Renin-angiotensin system	7	1	2	1	4	2	4	1	1	5	1	7	5	8
Replication, recombination and repair proteins	112549	101917	136827	124440	114943	100664	121100	89052	120368	124470	104107	103259	101640	96166
Restriction enzyme	26584	26537	22910	24534	26480	27743	26911	28709	29203	27954	27699	26180	27852	27868
Retinol metabolism	15751	16520	17422	17386	16552	16320	15954	15546	19083	20913	17507	17320	17105	16965
Rheumatoid arthritis	0	0	0	0	0	0	0	0	0	0	0	0	0	0
Riboflavin metabolism	40783	41185	42961	42884	42329	41237	42279	39909	48718	49170	44157	43483	43638	42353
Ribosome	302754	323325	337435	332495	316691	306177	310847	297886	375375	360335	318935	313041	318207	305258
Ribosome Biogenesis	181094	188645	194915	192754	187467	183512	188718	179714	214348	210568	191721	190612	191292	185495

Ribosome biogenesis in eukaryotes	5203	5598	6040	5921	5591	5350	5536	5051	6604	6472	5578	5619	5651	5404
Salivary secretion	0	0	0	0	0	0	0	0	0	0	0	1	1	0
Secondary bile acid biosynthesis	4096	4239	4080	3957	4163	4409	4534	4610	4170	3711	3957	3707	4048	4134
	23653	24359	24658	24853	24581	23976	24317	23396	27873	27269	24687	24309	24939	24122
Secretion system	1	8	1	3	6	5	1	1	5	9	7	5	9	1
Selenocompound metabolism	72047	71315	73367	73525	72978	71944	74921	70225	76412	77172	73643	73697	73334	72758
Sesquiterpenoid biosynthesis	4	26	62	69	30	40	23	0	48	34	38	44	40	48
Shigellosis	0	0	2	0	0	0	0	0	2	0	0	0	0	0
	11234	11144	11251	11283	11471	11417	11545	11288	12363	12261	11580	11301	11592	11443
Signal transduction mechanisms	2	8	8	0	5	7	6	7	9	7	8	3	6	9
Small cell lung cancer	108	252	292	351	209	52	32	28	679	293	131	193	232	71
Sphingolipid metabolism	10386	9707	10555	10382	10120	10336	12560	9985	10791	10299	10189	10527	10238	10316
Spliceosome	0	0	0	0	0	0	0	0	0	0	0	0	0	0
	45398	42386	39115	40683	44399	46005	45956	47639	41073	38096	42537	40875	43604	44591
Sporulation	2	5	9	6	8	9	8	3	5	0	3	1	1	3
Staphylococcus aureus infection	29555	27840	24126	25677	28357	30411	28779	32810	27574	24417	27314	24872	27909	28798
	21104	20266	19714	19992	21075	21414	22128	21630	21877	20616	20288	19397	20812	20752
Starch and sucrose metabolism	0	8	2	9	5	2	7	2	3	9	7	1	3	7
Steroid biosynthesis	4685	4701	4673	4767	4991	5015	4755	4968	6663	6714	5811	5779	5765	5514
Steroid hormone biosynthesis	276	445	852	859	592	521	492	153	1331	1841	921	1023	816	806
Stilbenoid, diarylheptanoid and gingerol biosynthesis	17607	16903	16263	16814	18040	18742	17912	18901	18583	17553	18146	17404	18422	18729
Streptomycin biosynthesis	24805	26423	34152	30668	27385	24548	28420	21418	36151	38457	29167	29682	27959	25742
Styrene degradation	9977	9567	9978	10367	10534	10712	10629	10049	10225	11125	11014	11442	11061	11412
Sulfur metabolism	73119	69822	68025	70157	72488	73386	75146	73415	75418	74923	74769	72629	73691	74113
Sulfur relay system	63859	62436	61513	63473	64519	64555	65552	64121	71984	72028	66725	64246	66150	65334
Synthesis and degradation of ketone bodies	12487	13089	14380	14724	13268	13089	13137	11267	11257	14491	13423	14713	13121	13958
Systemic lupus erythematosus	0	1	3	1	2	1	1	0	1	1	0	1	2	0
TGF-beta signaling pathway	0	0	0	0	0	0	0	0	0	0	0	0	0	0
Taurine and hypotaurine metabolism	28654	28285	27950	28329	29030	29372	29224	29209	29468	29025	28699	27906	28899	29090
Terpenoid backbone biosynthesis	77518	80956	85562	84108	81051	79218	80753	75713	91174	94009	83397	82988	82372	80670
Tetracycline biosynthesis	39215	38153	37285	38285	39413	39487	39210	39444	40341	40655	40295	39604	40090	40053
Thiamine metabolism	88076	86953	87985	88149	89095	89049	91893	88333	93537	92846	88845	86570	88858	88371

Tight junction	0	0	0	0	0	0	0	0	0	0	0	0	0	0
Toluene degradation	20145	20178	20981	21884	21389	21009	20710	19898	24900	24128	21792	21771	22112	21656
Toxoplasmosis	108	252	292	351	209	52	32	28	679	293	131	193	232	71
	43012	41252	40974	41379	42749	43493	44404	43673	42176	40651	41899	41030	42264	42710
Transcription factors	3	2	4	8	1	2	0	0	7	6	1	0	6	4
	14985	14973	14929	15200	15207	15131	15505	14893	16623	16283	15892	15947	15787	15558
Transcription machinery	7	9	2	5	7	0	1	9	0	4	9	9	0	0
Transcription related proteins	4374	3874	3775	3799	4243	4422	4393	4753	3916	3630	3977	3669	4087	4189
Translation factors	52600	57711	61520	60147	55743	53025	54670	50587	69599	68311	57704	57080	56855	53803
	11983	12368	12976	12824	12462	12018	12467	11626	14290	14389	12755	12691	12702	12240
Translation proteins	5	3	3	5	8	3	2	0	5	9	7	9	6	6
	14089	13369	13719	13749	14018	14090	14760	13997	14641	14296	14190	14125	14195	14138
Transporters	42	44	78	98	46	10	19	28	79	18	46	29	33	62
Tropane, piperidine and pyridine alkaloid biosynthesis	16319	15762	16715	17112	16820	16427	17385	15495	17976	18897	16931	17165	17034	16928
Tryptophan metabolism	89043	84214	88073	89893	92210	93054	93474	89295	87957	90813	91044	93025	92853	94887
Tuberculosis	17058	19706	22075	21211	19361	17301	17736	15759	29250	30280	22331	21873	21413	19068
	54393	52066	50235	51468	54353	55359	55908	56049	56334	54017	55346	53993	55555	55563
Two-component system	5	0	9	1	8	1	7	6	6	4	4	1	6	2
Type I diabetes mellitus	5341	5933	6445	6320	5728	5434	5527	5092	7722	7734	6185	5981	5948	5596
Type II diabetes mellitus	5445	6068	6721	6409	6013	5590	5693	5214	8318	8993	6822	6635	6533	6018
Tyrosine metabolism	69334	70496	76934	75357	72607	70719	72403	68180	78523	86802	75783	77212	74950	74052
Ubiquinone and other terpenoid-quinone biosynthesis	59541	62139	60957	63090	62914	61072	58725	60018	80313	79263	67435	65013	67237	63741
Ubiquitin system	8661	8143	7786	7958	8791	9079	8786	9446	9179	8552	8916	8542	9092	9090
VEGF signaling pathway	0	0	0	0	0	0	0	0	0	0	0	0	0	0
	11421	11314	11350	11572	11569	11565	11843	11403	12933	12908	11991	11701	11858	11729
Valine, leucine and isoleucine biosynthesis	3	3	0	0	0	2	7	2	2	0	0	3	2	2
	13612	13063	13569	13885	14034	14057	14142	13377	13210	13560	13576	13745	13798	14128
Valine, leucine and isoleucine degradation	6	2	4	2	0	0	5	6	7	1	4	4	9	3
Various types of N-glycan biosynthesis	1	0	1	0	0	0	0	0	8	0	0	0	0	0
Vascular smooth muscle contraction	0	0	0	0	0	0	0	0	0	0	0	0	0	0
Vasopressin-regulated water reabsorption	0	0	0	0	1	0	0	0	0	0	0	0	0	0

Vibrio cholerae infection	0	0	0	0	0	0	0	0	0	0	0	0	0	0
Vibrio cholerae pathogenic cycle	15024	15192	15014	15326	15488	15123	15331	14785	18392	18770	16569	15690	16096	15570
Viral myocarditis	108	252	292	351	209	52	32	28	679	293	131	193	232	71
Vitamin B6 metabolism	32831	32272	30858	31836	32651	33668	33875	33794	35405	34583	33329	31504	32972	33213
Wnt signaling pathway	0	0	0	0	0	0	0	0	0	0	0	0	0	0
Xylene degradation	9598	10092	10873	10626	10247	10403	10021	10165	11896	13268	12070	12333	11426	11513
Zeatin biosynthesis	5089	5520	5760	5680	5273	5112	5279	4972	6456	6208	5402	5219	5273	5066
alpha-Linolenic acid metabolism	5440	5489	5446	5774	5666	5654	5571	5214	5887	6509	5665	5753	5760	5837
beta-Alanine metabolism	60803	58006	61791	63022	63419	63756	65143	59598	61288	64904	62959	63882	63325	65056
beta-Lactam resistance	9043	8700	8146	8549	9066	9415	9069	9685	9510	9540	10105	9959	9821	9963
mRNA surveillance pathway	8	3	19	26	16	25	25	7	6	40	16	28	16	33
mTOR signaling pathway	0	0	0	0	0	0	0	0	0	0	0	0	0	0
p53 signaling pathway	109	254	293	353	210	53	33	28	679	293	131	194	232	72

Table S7. Fungi genera abundance during different fermentation stages of JZ22 and JB3.

OTU ID	JZ22 D0	JZ22 D2	JZ22 D4	JZ22 D6	JZ22 D8	JZ22 D10	JZ22 D12	JB3 D0	JB3 D2	JB3 D4	JB3 D6	JB3 D8	JB3 D10	JB3 D12
g__Cladosporium	28	0	0	0	0	0	1	185	0	0	0	0	0	0
g__Cochliobolus	11	0	0	0	0	0	0	100	0	0	0	0	0	0
g__Cryptosporidium	0	0	0	0	0	0	0	1	0	0	0	0	0	0
g__Galactomyces	0	0	0	0	0	0	0	2	0	0	0	0	0	0
g__norank_c__Agaricomycetes	10	0	0	0	0	0	0	21	0	0	0	0	0	0
g__norank_k__Fungi	1737	49	102	22	27	38	20	1917	24	38	97	53	62	71
g__norank_o__Malasseziales	4	0	0	0	0	0	0	11	0	0	0	0	0	0
g__norank_o__Tremellales	7	0	0	0	0	0	0	4	0	0	0	0	0	0
g__norank_p__Basidiomycota	3	0	0	0	0	0	0	0	0	0	0	0	0	0
g__norank_p__Chytridiomycota	2	0	0	0	0	0	0	0	0	0	0	0	0	0
g__norank_p__Glomeromycota	8	0	0	0	0	0	0	0	0	0	0	0	0	0
g__Oryza	30	0	0	1	0	0	0	48	0	2	0	1	1	1
g__Pichia	13410	89	60	54	39	80	90	19694	46	38	44	43	42	43
g__Saccharomycopsis	429	12	9	9	5	10	7	631	10	8	6	8	8	7
g__Symbiodinium	1	0	0	0	0	0	0	36	0	0	0	0	0	0
g__Trichosporon	2	0	0	0	0	0	0	11	0	0	0	0	0	0

g__unclassified_d__Eukaryota	4	0	0	0	0	0	0	0	0	0	0	0	0	0
g__unclassified_f__Colpodea	6	0	0	0	0	0	0	0	0	0	0	0	0	0
g__unclassified_f__Trichocomaceae	2158	45	22	18	11	35	26	1893	22	9	7	13	10	10
g__unclassified_o__Hypocreales	94	0	0	0	0	0	0	36	1	0	0	0	0	0
g__unclassified_o__Saccharomycetales	22659	40411	40415	40504	40526	40444	40463	16017	40505	40513	40453	40490	40486	40476
g__unclassified_p__Ascomycota	4	0	0	0	0	0	0	0	0	0	0	0	0	0

Microbial dynamics and metabolite changes in Chinese Rice Wine fermentation from sorghum with different tannin content

Jialiang Xu, Huijun Wu, Zhiwei Wang, Fuping Zheng, Xin Lu, Zhenpeng Li, Qing Ren

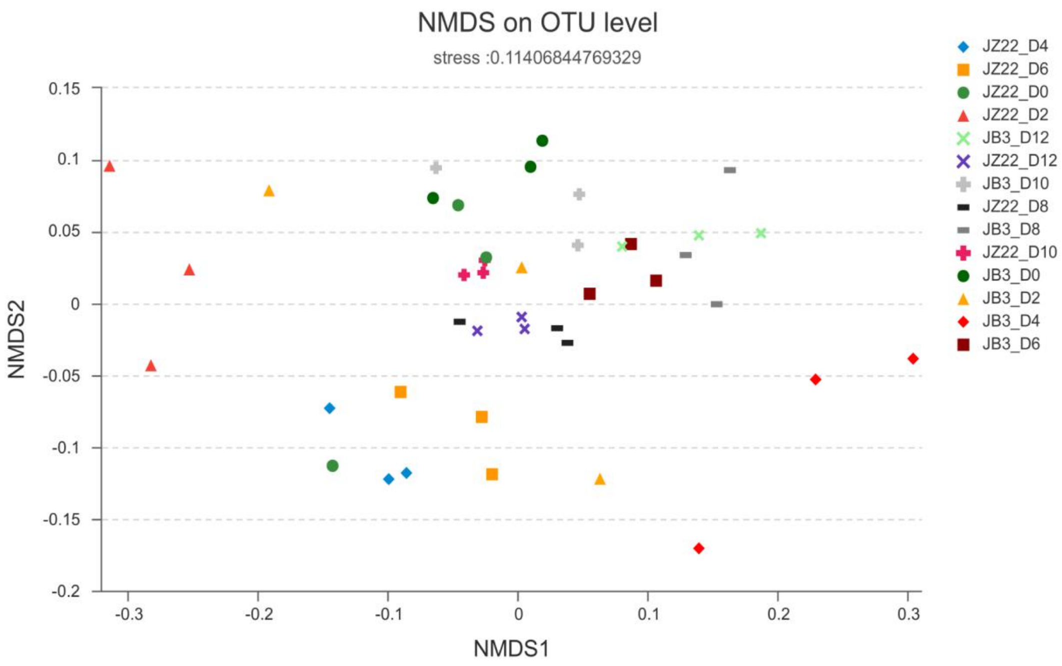

Figure S1. Non-metric Multidimensional Scaling (NMDS) distribution based on Weighted Unifrac distance.
